# *MiDaf16*-*like* and *MiSkn1-like* gene families are reliable targets to develop biotechnological tools for the control and management of *Meloidogyne incognita*

**DOI:** 10.1038/s41598-020-63968-8

**Published:** 2020-04-24

**Authors:** Marcos Fernando Basso, Isabela Tristan Lourenço-Tessutti, Reneida Aparecida Godinho Mendes, Clidia Eduarda Moreira Pinto, Caroline Bournaud, François-Xavier Gillet, Roberto Coiti Togawa, Leonardo Lima Pepino de Macedo, Janice de Almeida Engler, Maria Fatima Grossi-de-Sa

**Affiliations:** 1 Embrapa Genetic Resources and Biotechnology, Brasília-DF, 70297-400 Brazil; 2 Federal University of Brasília, Brasília-DF, 70910-900 Brazil; 3 Université de Grenoble Alpes, CNRS, CEA, INRA, 38054 Grenoble, Cedex 9 France; 4 UMR Institut Sophia Agrobiotech INRA/CNRS/UNS, Sophia Antipolis, France; 5 Catholic University of Brasília, Brasília-DF, 71966-700 Brazil

**Keywords:** Biotechnology, Molecular biology, Plant sciences

## Abstract

*Meloidogyne incognita* is a plant-parasitic root-knot nematode (RKN, PPN) responsible for causing damage to several crops worldwide. In *Caenorhabditis elegans*, the DAF-16 and SKN-1 transcription factors (TFs) orchestrate aging, longevity, and defense responses to several stresses. Here, we report that *MiDaf16-like1* and *MiSkn1-like1*, which are orthologous to DAF-16 and SKN-1 in *C. elegans*, and some of their targets, are modulated in *M. incognita* J2 during oxidative stress or plant parasitism. We used RNAi technology for the stable production of siRNAs *in planta* to downregulate the *MiDaf16-like1* and *MiSkn1-like1* genes of *M. incognita* during host plant parasitism. *Arabidopsis thaliana* and *Nicotiana tabacum* overexpressing a hairpin-derived dsRNA targeting these genes individually (single-gene silencing) or simultaneously (double-gene silencing) were generated. T_2_ plants were challenged with *M. incognita* and the number of eggs, galls, and J2, and the nematode reproduction factor (NRF) were evaluated. Our data indicate that *MiDaf16-like1*, *MiSkn1-like1* and some genes from their networks are modulated in *M. incognita* J2 during oxidative stress or plant parasitism. Transgenic *A. thaliana* and *N. tabacum* plants with single- or double-gene silencing showed significant reductions in the numbers of eggs, J2, and galls, and in NRF. Additionally, the double-gene silencing plants had the highest resistance level. Gene expression assays confirmed the downregulation of the *MiDaf16-like1* and *MiSkn1-like1* TFs and defense genes in their networks during nematode parasitism in the transgenic plants. All these findings demonstrate that these two TFs are potential targets for the development of biotechnological tools for nematode control and management in economically important crops.

## Introduction

Plant-parasitic nematodes (PPNs) are one of the major agricultural pathogens worldwide^1^. PPNs disturb plant roots by altering the cell cycle, increasing the size of parasitized cells, and causing cell hyperproliferation. This process results in the compatible interaction and development of nematode feeding sites (galls)^2–6^, which disrupt the uptake of water and nutrients and reduce plant growth and yield^7–9^. Root-knot nematodes (RKNs) are obligate sedentary endoparasites from the genus *Meloidogyne* spp.^10^. *Meloidogyne incognita* is one of the most commonly reported species, causing damage in several crops of economic importance worldwide^11^. Its life cycle comprises of six stages, namely, egg, J1 (first-stage juvenile), J2 (second-stage juvenile), J3 (third-stage juvenile), J4 (fourth-stage juvenile), and adults (female and male). The J3, J4, and females are typically sedentary endophytes, while the eggs and J2 are exophytes^11^. The limited range of available control agents or resistant cultivars has limited the efficiency of nematode control and management^1,12^. Thus, the development of new biotechnology tools is of great importance to overcome these challenges.

Plant-nematode interactions involve an extensive molecular immunity network involved in both defense and counter-defense^13^. After recognition of PPN elicitors, the host plants increase the production of reactive oxygen and nitrogen species (*e.g*., hydrogen peroxide-H_2_O_2_), and other toxic molecules derived from secondary metabolism^14–17^. In contrast, PPNs increase the production and release of antioxidant and detoxifying compounds (*e.g*., ROS scavengers, glutathione peroxidases, peroxidases, peroxiredoxins, and catalases)^18–20^, and effector proteins to overcome the host defense^21–23^. Thus, nematodes minimize plant cell damage, develop a feeding site, and promote cellular reproduction in susceptible plants^24,25^.

In *Caenorhabditis elegans*, the insulin/IGF-1 signaling (IIS) pathway acts as a major mediator between nematode development and stress responses^26^. The IIS pathway modulates oxidative stress responses by activating the phosphorylation cascade of PDK-1/AKT proteins, leading to the sequestration of DAF-16 and SKN-1 transcription factors (TFs) in the cytoplasm^27,28^. The *C. elegans* DAF-2 protein acts as a membrane receptor for insulin, which negatively regulates DAF-16 and SKN-1^29,30^. In contrast, miR-71 expression inhibits this phosphorylation cascade, allowing the nuclear translocation of DAF-16 and SKN-1^31,32^. *Daf-16* and *Skn-1*TFs are large families of genes containing a highly conserved DNA binding region (FOXO and bZIP domains, respectively). These TFs are responsible for the modulation of up to 500 and 846 genes, respectively, which code for numerous protein families with some overlapping targets in the nematode secretome^13^. These target proteins are implicated in the regulation of the antioxidant and detoxification pathways and the unfolding protein response^33–37^. The genome and transcriptome sequence data from PPNs revealed that the orthologues of the *Daf-16* and *Skn-1* genes from *C. elegans* are also present in *M. incognita*^11,38,39^. Thus, it is postulated that DAF-16 and SKN-1 can also orchestrate some important immune adaptive responses to environmental stresses in PPNs during both oxidative stress and plant parasitism^13,19,20^. These features suggest that the *Daf-16* and *Skn-1* genes are interesting targets for the development of RNA interference (RNAi)-based new biotechnological tools (NBTs) for nematode control.

Herein, working with orthologous genes, we used RNAi technology for the *in planta* production of engineered siRNAs to downregulate the *MiDaf16-like1* and *MiSkn1-like1* genes of *M. incognita*. The *A. thaliana* and *N. tabacum* overexpressing a dsRNA that targets these genes were generated. Then, T_2_ plants were challenged with *M. incognita*, and the resistance level of these plants was evaluated. Our results showed that both the *MiDaf16-like1* and *MiSkn1-like1* genes and some other genes downstream of these TFs are upregulated in *M. incognita* J2 during oxidative stress or plant parasitism. Single- or double-gene silencing plants of *A. thaliana* or *N. tabacum* showed significant reductions in the number of eggs, J2, and galls, and a decrease in the nematode reproduction factor (NRF) compared with wild-type (WT) plants. The double-gene silencing plants had the highest resistance level, which was correlated with efficient downregulation of the two TFs, and lower expression of genes involved in oxidative stress response, detoxification, and the antioxidant pathway. As a result of our study, we have characterized for the first time the functions of DAF-16 and SKN-1 TFs in the PPN *M. incognita*.

## Results

### *In silico* analysis reveals potential *Daf-16* and *Skn-1* orthologous genes in *M. incognita*

**Figure 1:**
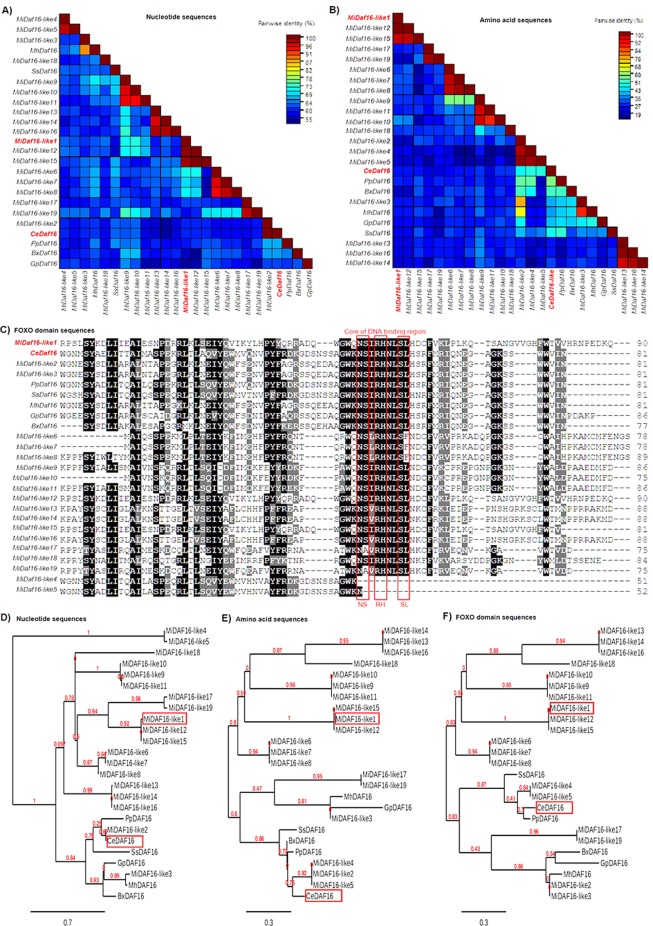
*In silico* analysis of *Daf-16* (Dauer Formation-16) genes from nematodes. Pairwise sequence identity matrix from nucleotide (**A**) and amino acid (**B**) sequences generated using the Sequence Demarcation Tool Version 1.2 software^68^. (**C**) Positional conservation of the FOXO (Forkhead box) domain generated from multiple sequence alignment by the Color Align Conservation software^69^. Evolutionary analysis from nucleotide (**D**), amino acid (**E**) and FOXO domain (**F**) sequences generated from the Phylogeny.fr web service^71^. *M. incognita* (Mi), *C. elegans* (Ce), *P. pacificus* (Pp), *M. hapla* (Mh), *G. pallida* (Gp), *S. stercoralis* (Ss) and *B. xylophilus* (Bx). *MiDaf16-like1* (Minc3s02528g30466), *MiDaf16-like2* (Minc3s00293g09565), *MiDaf16-like3* (Minc3s06738g40249), *MiDaf16-like4* (Minc3s02143g28529), *MiDaf16-like5* (Minc3s03756g34708), *MiDaf16-like6* (Minc3s06700g40200), *MiDaf16-like7* (Minc3s00670g15892), *MiDaf16-like8* (Minc3s00896g18634), *MiDaf16-like9* (Minc3s05371g38122), *MiDaf16-like10* (Minc3s01745g26020), *MiDaf16-like11* (Minc3s01171g21384), *MiDaf16-like12* (Minc3s00913g18806), *MiDaf16-like13* (Minc3s00459g12679), *MiDaf16-like14* (Minc3s09607g43370), *MiDaf16-like15* (Minc3s03624g34358), *MiDaf16-like16* (Minc3s00600g14903), *MiDaf16-like17* (Minc3s01319g22739), *MiDaf16-like18* (Minc3s00100g04542), *MiDaf16-like19* (Minc3s02176g28694), *MhDaf16* (contig353.frz3.gene4), *SsDaf16* (AAQ23177), *CeDaf16* (R13H8.1c), *PpDaf16* (AGA16632), *BxDaf16* (BXY_0566400), and *GpDaf16* (GPLIN_001276900).

**Figure 2:**
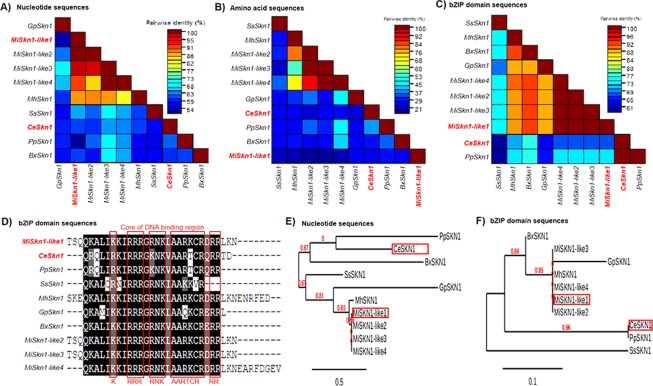
*In silico* analysis of *Skn-1* (Skinhead-1) genes from nematodes. Pairwise sequence identity matrix from nucleotide (**A**), amino acid (**B**) and Basic leucine zipper (bZIP) domain (**C**) sequences generated using Sequence Demarcation Tool version 1.2 software^68^. (**D**) Positional conservation of the bZIP domain generated from multiple sequence alignment by Color Align Conservation software^69^. Evolutionary analysis from nucleotide (**E**) and bZIP domain (**F**) sequences generated from the Phylogeny.fr web service^71^. *M. incognita* (Mi), *C. elegans* (Ce), *P. pacificus* (Pp), *M. hapla* (Mh), *G. pallida* (Gp), *St. stercoralis* (Ss) and *B. xylophilus* (Bx). *MiSk1-like1* (Minc3s02028g27861), *MiSkn1-like2* (Minc3s02028g27862), *MiSkn1-like3* (Minc3s08604g42418), *MiSkn1-like4* (Minc3s03116g32841), *MhSkn1* (MhA1_Contig1686.frz3.gene3), *SsSkn1* (SSTP_0000496400), *CeSkn1* (CCD62212), *PpSkn1* (PPA13755), *BxSkn1* (BXY_0018200), and *GpSkn1* (GPLIN_000599400).

The DAF-16 and SKN-1 TFs were first identified in *C. elegans* and associated with the defense system against oxidative stress and increased nematode lifespan^40–42^. The family of these TFs is considered highly conserved not only in nematodes but also in mammals^34,43,44^. We used *in silico* analyses to search for putative orthologous genes in the *M. incognita* genome and identified 19 *Daf-16-*like and 4 *Skn-1-*like genes (Supplemental Table 1). The *Daf-16* orthologous (designated *MiDaf16-like1* to 19) genes showed low nucleotide (Fig. 1A) or amino acid (Fig. 1B) sequence identity when compared with those the *C. elegans*, other phytonematodes (*Meloidogyne hapla*, *Globodera pallida* and *Bursaphelenchus xylophilus*), the free-living nematode *Pristionchus pacificus* and the human parasite *Strongyloides stercoralis*. However, the FOXO domain, which corresponds to the core of the DNA binding region, was highly conserved in almost all 19 *MiDaf16-like* genes, with the exception of the *MiDaf16-like4* and 5 genes (Fig. 1C). A phylogenetic analysis using nucleotide sequences of these *MiDaf16-like* genes suggested that *MiDaf16-like2* could be considered an orthologue of the *Daf-16* gene from *C. elegans* (Fig. 1D). However, phylogenetic analyses using the full amino acid sequence (Fig. 1E) or only the FOXO domain (Fig. 1F) sequences do not clearly show that the *MiDaf16-like2* gene can unambiguously be considered the orthologue. In contrast, the *MiSkn1-like1* to 4 genes showed greater identity in their nucleotide (Fig. 2A), amino acid (Fig. 2B), or only bZIP domain (Fig. 2C) sequences among themselves or when compared with sequences from other phytonematodes, but they had low sequence identity with the *Skn-1* gene of *C. elegans*. Similar to the *MiDaf16-like* genes, the core of the DNA binding region of the bZIP domain from the *MiSkn1-like* genes was highly conserved compared with those of *C. elegans* and other phytonematodes (Fig. 2D). Phylogenetic analyses using nucleotide (Fig. 2E) or only the bZIP domain (Fig. 2F) sequences showed that the *MiSkn1-like1* to 4 genes are clustered in a different clade from the *Skn-1* gene of *C. elegans*. Thus, among multiple orthologous putative genes in *M. incognita*, we designated *MiDaf16-like1*, 2, 3, 11, and 12 as potential orthologues of the *Daf-16* gene of *C. elegans*, while for the *MiSkn1-like* genes, it was not evident that may be considered the orthologue of the *Skn-1* gene of *C. elegans*.

### *MiDaf16-like1* and *MiSkn1-like1* expression are modulated during oxidative stress and plant parasitism

**Figure 3:**
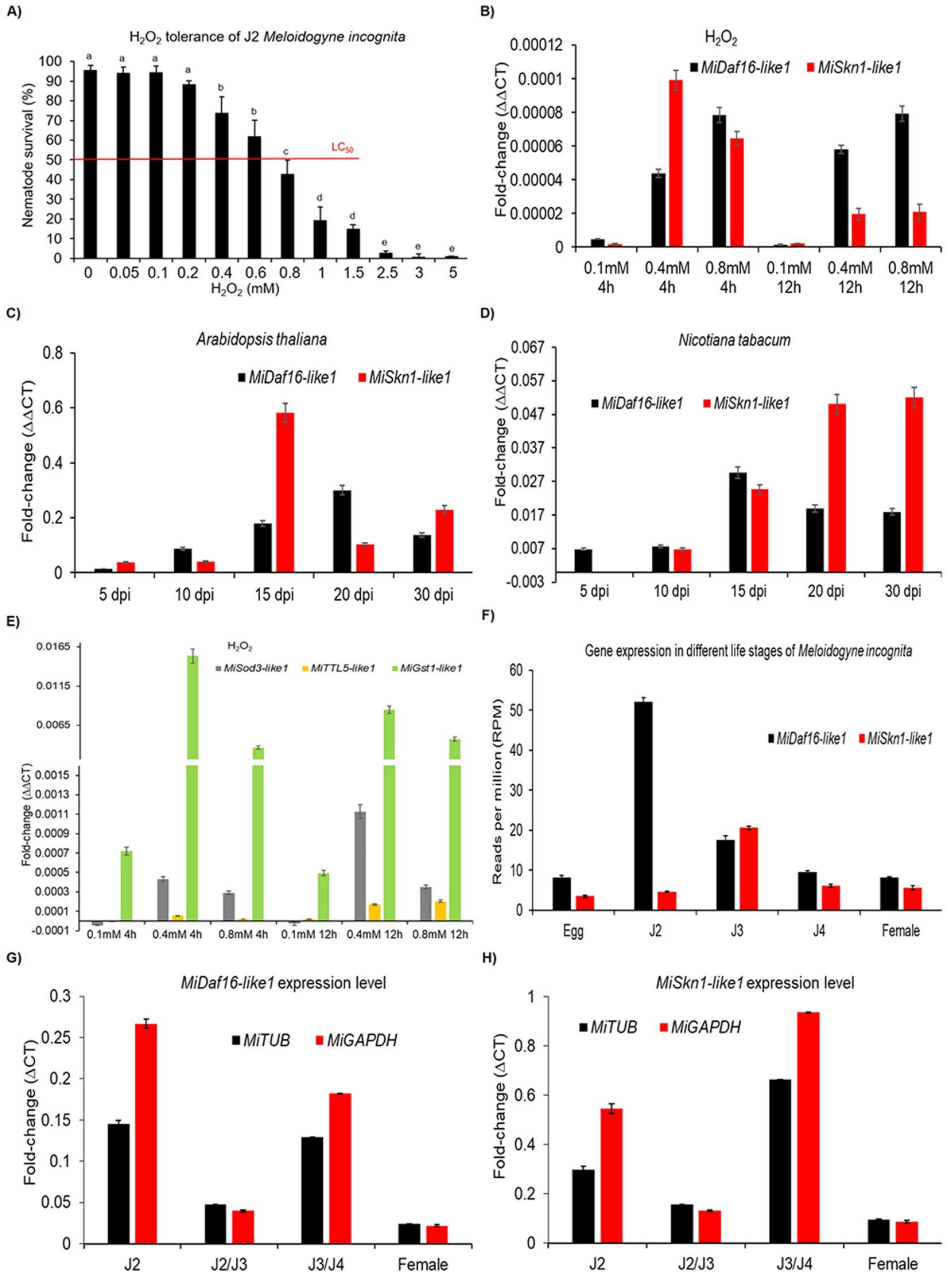
Expression profiles of *MiDaf16-like1* (Minc3s02528g30466) and *MiSkn1-like1* (Minc3s02028g27861) in response to oxidative stress and plant parasitism. (**A**) Survival percentage (%) of *M. incognita* J2 race 3 after overnight exposure to different concentrations of H_2_O_2_, as described by Vicente *et al*.^61^. Error bars represent confidence intervals corresponding to three biological replicates. Different letters above the columns indicate significant differences (p-value <0.05) between different survival percentages in each H_2_O_2_ treatment, according to Tukey’s test. (**B**) Expression profile of *MiDaf16-like1* and *MiSkn1-like1* in *M. incognita* J2 race 3 after 4 and 12 h of exposure to different concentrations of H_2_O_2_. (**C**) Expression profile of *MiDaf16-like1* and *MiSkn1-like1* during *M. incognita* parasitism (5 to 30 days post-inoculation; dpi) in (**C**) *A. thaliana* and (**D**) *N. tabacum*. (**E**) Expression profile of *MiSod3-like1*, *MiTTL5-like1*, and *MiGst1-like1* during exposure of *M. incognita* J2 race 3 to H_2_O_2_. Newly hatched J2 were used as controls for all assays of gene expression level. Error bars represent confidence intervals corresponding to three biological replicates. (**F**) Expression profiles of *MiDaf16-like1* and *MiSkn1-like1* in different life stages of *M. incognita* using transcriptome datasets (BioProject number: PRJNA390559^39^); retrieved from the BioSample database (NCBI). Error bars represent confidence intervals corresponding to three libraries per life stage of the nematode. Expression profile measured by real-time PCR of the *MiDaf16-like1* (**G**) and *MiSkn1-like1* genes in different life stages of *M. incognita* race 3. The relative expression levels were normalized with the *β-tubulin* (*MiTUB*) and *glyceraldehyde 3-phosphate dehydrogenase* (*MiGAPDH*) endogenous reference genes (Supplemental Table 3). Error bars represent confidence intervals corresponding to three biological replicates.

To confirm the association of these two TFs in the defense response of *M. incognita* against oxidative stress and plant parasitism, we selected *MiDaf16-like1* and *MiSkn1-like1* for further study. Initially, we determined that 0.8 mM H_2_O_2_ is the lethal concentration (LC_50_) for newly hatched *M. incognita* J2 (Fig. 3A). Then, we exposed 5,000 newly hatched *M. incognita* J2 to 0.1 mM, 0.4 mM, and 0.8 mM H_2_O_2_ for 4 and 12 hours, collected them, isolated the RNA, and evaluated the expression profile of these two genes over time in response to the different concentrations of H_2_O_2_, compared to newly hatched J2 maintained in Milli-Q water. Real-time quantitative PCR assays showed that both genes were in fact modulated in response to oxidative stress (Fig. 3B). The *MiDaf16-like1* gene was gradually upregulated in J2 exposed from 0.1 mM to 0.8 mM H_2_O_2_, maintaining a constant expression level over time. In contrast, the *MiSkn1-like1* gene was highly upregulated in J2 exposed to 0.4 mM H_2_O_2_ for 4 h, while its expression level decreased after 12 h of exposure. Regarding the expression profile in the preparasitic phase or during the parasitism of host plants, we observed that these two genes are also modulated throughout the parasitic cycle of the nematode in both *A. thaliana* (Fig. 3C) and *N. tabacum* (Fig. 3D) WT plants. The highest level of expression of both genes was observed after 15 days post-inoculation (dpi), showing that they can act simultaneously in the defense process of *M. incognita* against multiple stresses during the parasitic phase. Then, we evaluated the expression profiles of some genes in the networks of these two TFs. Among them, the *MiSod3-like1*, *MiGst1-like1*, and *MiTTL5-like1* genes, which potentially act in the *M. incognita* antioxidant pathway, in the detoxification pathway, and as an effector secreted by the nematode in response to oxidative stress, respectively. Real-time PCR assays showed that these genes are also modulated in *M. incognita* J2 when they are exposed to oxidative stress (Fig. 3E). Here, we again used H_2_O_2_ as the oxidative stress-inducing agent and exposed newly hatched *M. incognita* J2 to this stress at different concentrations and exposure times. The *MiSod3-like1* and *MiGst1-like1* genes had higher expression than *MiTTL5-like1*, which increased in parallel with the increasing H_2_O_2_ concentrations and the time of exposure of the nematode. These findings provide evidence that the *MiDaf16-like1* and *MiSkn1-like1* genes are indeed upregulated in *M. incognita* under oxidative stress, and that these genes modulate defense genes in the nematode. Next, we evaluated the expression profiles of these two TFs in the different life stages of *M. incognita*. Initially, we mined transcriptome datasets available in a public database (NCBI SRA) generated from the egg mass, J2, J3, J4, and *M. incognita* females^39^. These *in silico* results showed that the two genes are potentially expressed in all life stages of the nematode, with the *MiDaf16-like1* gene having the highest number of reads (RPM) mapped in the J2 stage, while the *MiSkn1-like1* gene had the largest number of reads mapped in stage J3 (Fig. 3F). In addition, we used these same transcriptome datasets to evaluate the expression profiles of all 19 *MiDaf16-like* and 4 *MiSkn1-like* genes in the different life stages of *M. incognita*. Our *in silico* results showed that all the *MiDaf16-like* genes are potentially expressed at all life stages of *M. incognita*, except *MiDaf16-like7*, for which no reads were mapped to its transcript (Supplemental Fig. 1A to E). The expression profiles of these genes were differentially modulated throughout the life cycle of the nematode, especially *MiDaf16-like1*, 2, 12, 16, and 19, which had more reads mapped to their transcripts, suggesting that they had a higher level of expression than the other genes. In addition, the highest level of expression of these genes was observed at the J2 stage, whereas the *MiDaf16-like1* gene had a similar expression profile at all life stages, except for the J2 stage, which had a 5-fold greater number of reads mapped to its transcript than the other stages, suggesting a higher expression level in this stage. Regarding the *MiSkn1-like* genes, the four genes also showed potential expression throughout the life cycle of *M. incognita*, with greater expression in the J2 and J3 stages (Supplemental Fig. 1F). The *MiSkn1-like2* gene had the highest number of reads mapped to its transcript compared to the others overall, whereas *MiSkn1-like1* had the highest level of reads mapped to its transcript in the J3 stage. Real-time PCR assays using the total RNA isolated from the different plant-parasitic stages of *M. incognita* showed that the expression profiles of these two genes are indeed upregulated throughout the cycle of parasitism. The *MiDaf16-like1* gene showed a greater expression level in the J2 stage (Fig. 3G), while the *MiSkn1-like1* gene had a greater expression level in the J3/J4 stage (Fig. 3H). Finally, our results confirm that these genes are indeed associated with the defense response to oxidative stress and plant parasitism. In addition, their expression profile is orchestrated throughout their entire life cycle and in all phases of plant parasitism.

### Downregulation of the *MiDaf16-like1* and *MiSkn1-like1* genes by RNAi

**Figure 4:**
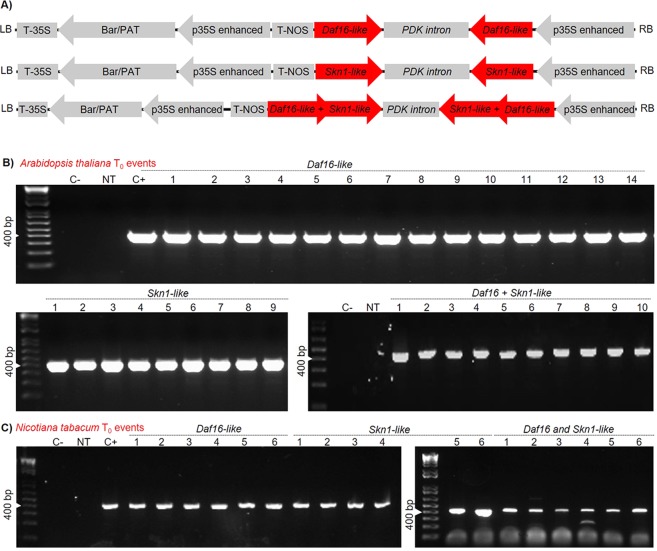
Binary vectors and genetic transformation of *A. thaliana* and *N. tabacum*. (**A**) Overview of the binary vectors for single knockdown of *MiDaf16-like1* or *MiSkn1-like1*, and both simultaneously (double-gene silencing), which were used for *in planta* transformation mediated by *Agrobacterium tumefaciens*. (**B**) PCR detection of the transgenes in *A. thaliana* (**B**) and *N. tabacum* (**C**) T_0_ plants. Marker: 1.0-kb DNA ladder (Invitrogen^®^ Cat. #10787018); NT: non-transgenic line used as a negative control for the PCR assay and bioassays in the greenhouse; C+: a transgenic plant used as a positive control for the PCR assay.

**Figure 5:**
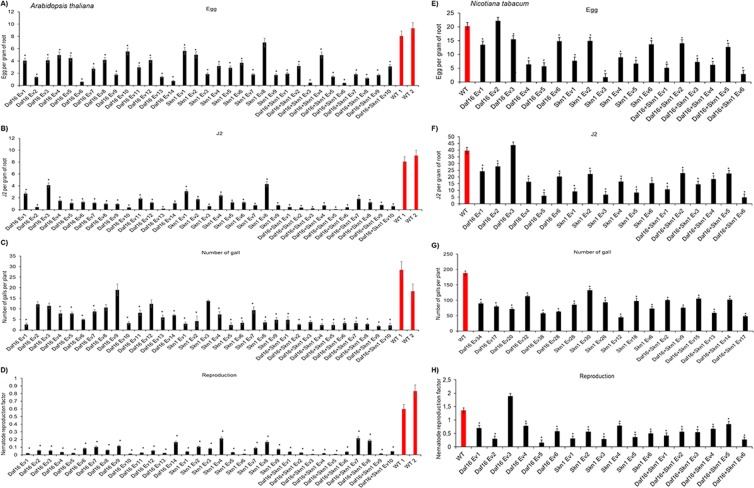
Evaluation of the resistance levels of *A. thaliana* (**A** to **D**) and *N. tabacum* (**E** to **H**) T_2_ plants to infection with *M. incognita* race 3. The number of eggs and J2 per gram of root, number of galls per plant, and NRF were evaluated 60 days post-inoculation. Error bars represent confidence intervals corresponding to three technical replicates. Asterisks indicate significant differences based on Tukey’s test at 5%.

To evaluate the effect of downregulation of the *MiDaf16-like1* and *MiSkn1-like1* genes in the phytopathogenicity of *M. incognita*, we generated several transgenic plants of *A. thaliana* and *N. tabacum* by overexpressing an engineered dsRNA to accumulate siRNAs in these plants. These dsRNAs were engineered to downregulate *MiDaf16-like1* or *MiSkn1-like1* (single-gene silencing), or both genes simultaneously (double-gene silencing) (Fig. 4A). For the modulation of the *MiDaf16-like1* gene, the dsRNA was constructed with a nucleotide sequence from the FOXO domain of this gene (Supplemental File 1), while for the modulation of the *MiSkn1-like1* gene, we used a sequence from the bZIP domain (Supplemental File 2). Because we used a relatively conserved region for each of these two genes (Figs. 1C and 2D) to direct the *in planta* production of siRNAs, the *MiDaf16-like12* and 15 (Supplemental Fig. 2A), and *MiSkn1-like 2* to 4 (Supplemental Fig. 2B) genes could also be potentially downregulated during parasitism in these plants. In *A. thaliana*, we generated 14 independent transformants to drive the downregulation of the *MiDaf16-like1* gene, nine transformants for downregulation of the *MiSkn1-like1* gene, and ten double-gene silencing transformants for simultaneous downregulation of these genes (Fig. 4B). In contrast, six independent transformants were generated for each of these strategies (single- or double-gene silencing) in *N. tabacum* (Fig. 4C). Then, *A. thaliana* and *N. tabacum* plants of the T_2_ generation were challenged to evaluate their resistance to *M. incognita*. At 60 dpi of freshly hatched J2, the number of eggs, J2, and galls, and the NRF were determined. In almost all single- or double-gene silencing plants of *A. thaliana*, we observed a significant reduction in the number of eggs (Fig. 5A), J2 (Fig. 5B), and galls (Fig. 5C), and NRF (Fig. 5D) ranging from 20 to 80%, when compared to that of WT plants. In addition, the double-gene silencing plants presented an apparently better performance than the single-gene silencing plants (Supplemental Fig. 3A). In addition, we evaluated some of these same *A. thaliana* single- and double-gene silencing plants for resistance to the *M. incognita* J2 strain Morelos (Mexican isolate). Interestingly, we observed a high resistance level similar to that when inoculated with *M. incognita* race 3 with respect to the numbers of both galls (Supplemental Fig. 3D) and egg mass (Supplemental Fig. 3E). In the *N. tabacum* plants, we also observed reductions in the number of eggs (Fig. 5E), J2 (Fig. 5F), and galls (Fig. 5G), and in the NRF (Fig. 5H) in most transgenic plants compared to WT plants. Similar to the *A. thaliana* plants, the *N. tabacum* double-gene silencing plants showed an apparently better performance than the single-gene silencing plants (Supplemental Fig. 3B). Additionally, we evaluated the morphology of galls at 45 dpi in WT, single-gene silencing plants, and double-gene silencing plants of *A. thaliana*. In the WT line, we observed the end of the nematode life cycle, while the single-gene silencing lines showed giant cells lacking the typical dense cytoplasm that were apparently smaller and an apparent delay in nematode development. Similarly, the double-gene silencing lines showed giant cells lacking the characteristic dense cytoplasm but were apparently smaller (Supplemental Fig. 3C). Overall, our findings confirm that the potential *in planta* downregulation of the *MiDaf16-like1* and *MiSkn1-like1* genes disrupts the efficiency of *M. incognita* in parasitizing the host plant.

### The *M. incognita* susceptibility is correlated with downregulation of its defense genes

To confirm whether transgenic plant parasitism results in siRNA uptake and the consequent downregulation of *MiDaf16-like1* and *MiSkn1-like1*, we harvested galls at 60 dpi on WT and transgenic lines, isolated total RNA and evaluated the expression profiles of these genes. In addition, we evaluated the expression profiles of some genes from the DAF-16 and SKN-1 networks involved in nematode defense against stresses. Real-time PCR assays showed that *MiDaf16-like1* (Supplemental Fig. 5A), *MiSkn1-like1* (Supplemental Fig. 5B), or both simultaneously (Supplemental Fig. 5C) were downregulated in *M. incognita* during parasitism in single- or double-gene silencing plants, respectively. In addition, we observed that at least some defense genes from the *Daf-16* and *Skn-1* networks were consequently also downregulated during the parasitism of nematodes in single- or double-gene silencing plants (Supplemental Fig. 5D). In *M. incognita* infecting both single- or double-gene silencing plants, the *MiPRDX2-like1*, *MiSod3-like1*, *MiGPX-like1*, *MiGst1-like1*, and *MiSod1-like1* genes were simultaneously downregulated at a level of approximately 80%, with the exception of the *MiGst1-like1* gene in one of the double-gene silencing plants. The *in silico* analyses using the same transcriptome datasets available in the public database (NCBI SRA) generated from the egg mass, J2, J3, J4, and females of *M. incognita*^39^ showed that these defense genes were differentially modulated in all life stages of the nematode (Supplemental Fig. 4A to F). Given this, our findings confirm the efficient uptake of siRNAs during plant parasitism and downregulation of these TFs and genes in their networks, resulting in a decreased ability of *M. incognita* to infect host plants.

## Discussion

The insulin/IGF-1 signaling (IIS) pathway was first shown to regulate dauer formation and resistance to multiple stresses in *C. elegans*^26,45^. Subsequently, the IIS pathway was also characterized in other organisms (*e.g*., PPN, *Drosophila melanogaster*, mice, and humans), confirming its role associated with the aging, longevity and defense response to stresses^43,46^. In *C. elegans*, *Daf-2* and *Age-1* genes coding to IGF-1 insulin receptor and phosphatidylinositol-3-OH kinase (PI3K) of the IIS pathway, respectively. The inhibition of these two genes results in lifespan extension in the nematode^30,47,48^. The signal transduction in the IIS pathway is orchestrated by sequential events and modulated by environmental conditions (*e.g*., high insulin content, no stresses, oxidative stress, stress from the environment). In favorable conditions (*e.g*., high insulin content and no stresses), the IIS pathway is activated, resulting in the normal development of nematodes. The binding of insulin or insulin-like peptides to the DAF-2 receptor results in the activation of the AGE-1 and PI3K genes, increasing the level of phosphatidylinositol(3,4,5)-trisphosphate (PIP3). PIP3 accumulation is balanced by DAF-18/PTEN phosphatase, promoting its conversion to phosphatidylinositol(4,5)-bisphosphate (PIP2). Then, PIP3 activates the kinase signaling cascade, composed of 3-phosphoinositide-dependent protein kinase 1 (PDK-1), protein kinase B (AKT-1 and -2), and serum- and glucocorticoid-inducible kinase-1 (SGK-1). In turn, these components phosphorylate and inactivate the DAF-16 and SKN-1 TFs by sequestering them in the cytoplasm and preventing nuclear import^29^. In unfavorable conditions to the nematode (*e.g*., low insulin content, oxidative stress, adverse environment), the IIS pathway is deactivated, resulting in DAF-16 and SKN-1 TF activation by efficient translocation from the cytoplasm to the nucleus. In addition, miR-71 inhibits the phosphorylation cascade via posttranscriptional regulation of the *Age-1* and *Akt* genes, allowing the efficient translocation of DAF-16 and SKN-1 to the nucleus^31^. In the oxidative stress response, the PMK-1 kinase from the p38 MAPK pathway phosphorylates SKN-1, leading to its translocation and nuclear accumulation^49,50^. On the other hand, the 14-3-3 scaffolding proteins bind to the phosphorylation sites of DAF-16 and contribute to its sequestration within the cytoplasm^51,52^. In the nucleus, the DAF-16 and SKN-1 TFs are responsible for the transcriptional activation of up to 500 and 846 genes, respectively^13,53^. These activated genes belong to several functional groups and are implicated in the aging and longevity process and the antioxidant, detoxification, and protein unfolding response pathways^54–57^. Thus, downregulation of the IIS pathway and the consequent upregulation/activation of the DAF-16 and SKN-1 TFs confer high resistance to a variety of stresses (*e.g*., heat, hypoxia, osmotic, UV, metal toxicity, and oxidative stresses) in *C. elegans*.

Genome and transcriptome studies from PPNs have revealed genes orthologous to *Daf-16* and *Skn-1* from *C. elegans* in the *Meloidogyne*, *Pratylenchus*, and *Bursaphelenchus* genera^39,58–60^. The high level of sequence conservation suggests similar functions to those observed in *C. elegans*^13,35,59^. Studies of comparative genomes in PPNs have identified numerous defense genes from the DAF-16 and SKN-1 networks involved in ROS scavenging, coding proteins linked to the antioxidant pathway, superoxide dismutases (SOD), catalases (CAT), glutathione S-transferases (GST), glutathione peroxidase (GPX), and peroxiredoxins (PRDX)^11,18,20,58,61^. In addition, plant-nematode interaction studies suggest the involvement of these genes in the nematode defense response, both in the control of endogenous oxidative stress and in the modulation of the host cell^18,20,62^. In this study, we identified at least 19 *MiDaf16-like* and 4 *MiSkn1-like* genes in the *M. incognita* genome, with a high degree of sequence conservation in the FOXO and bZIP domains, and almost all were expressed in all life stages of the nematode. In addition, we confirmed that the *MiDaf16-like1* and *MiSkn1-like1* genes are expressed in all life stages of *M. incognita* and are modulated in response to oxidative stress and during plant parasitism, confirming the IIS pathway activation and its functionality in *M. incognita*; we also showed that some genes in their networks are consequently also modulated. Interestingly, without targeting all the members of these families because they are highly conserved in nematodes and other animals, these two gene families may be potential targets for the development of NBTs to impair the defense pathways of PPNs during plant parasitism. *M. incognita* is a RKN^10^, an important plant pathogen causing economic losses in several crops worldwide^11^, and an excellent model system for obligate sedentary endoparasitic PPNs^13,21^. Our findings confirm that *in planta* downregulation of *MiDaf16-like1*, *MiSkn1-like1* (single-gene silencing), or both genes simultaneously (double-gene silencing), makes nematodes more susceptible to stress conditions during parasitism, significantly reducing the number of galls and eggs, NRF, and, consequently, decreasing the source of inoculum. In addition, we also found that the nematodes maintained in transgenic plants with either single- or double-gene silencing constructs present an apparent delay in nematode development, while their giant cells are smaller. These results can be explained by a depletion of nematode defense or counter-defense mechanisms during plant parasitism and potentially by a disruption of the fine-tuning between the nematode defense and development pathways. In agreement with this hypothesis, we have confirmed that downregulation of the *MiDaf16-like* and *MiSkn1-like1* genes results in strong downregulation of genes from their networks during parasitism of *M. incognita* in single- or double-gene silencing plants. In this respect, the two superoxide dismutases (*MiSod1-like1* and *MiSod3-like1*), peroxiredoxin (*MiPRDX2-like1*), glutathione S-transferase and peroxidase (*MiGst1-like1* and *MiGPX-like1*), which act in the nematode antioxidant and detoxification pathways^18–20,63,64^, were indeed downregulated in *M. incognita* during parasitism in transgenic plants.

Recently, Koutsovoulos *et al*.^65^ sequenced the genomes of 11*M. incognita* isolates race 1 to 4 infecting different host plant across Brazil (including the race 3, used in this study) and showed that cumulative fixed divergence across these Brazilian isolates and the reference genome (*M. incognita* strain Morelos) reached approximately 0.02% of the nucleotides. However, these few point variations between the isolates showed no significant association with the host species, geographical origin of the samples and crop on which they were collected, and there were no disruptive variations identified in the coding regions of genes. Thus, these authors suggested that other factors are more important for the adaptation of this species than these few point mutations. Consistent with this hypothesis, Castagnone‐Sereno *et al*.^66^ showed that convergent gene copy number variations (CNV) were associated with breaking down of resistance by *M. incognita*. In addition, these authors showed that CNV and speculated that expression levels of these genes are two major features associated with *M. incognita* pathogenicity in different hosts. This data suggests that *MiDAF16-like* and *MiSKN1-like* gene families may vary in copy number of these genes between different *M. incognita* isolates and races, or host crops. In contrast, these genes may present considerable sequences similarity between different nematode races or isolates. Thus, the use of this biotechnological strategy is of great relevance in crops or cultivars of economic interest (such as soybean, cotton, coffee, cocoa, and tomato, among others) considered to be susceptible to this nematode or cultivated in areas with a high incidence of this pathogen. In addition, these two gene families have great potential to be modulated by NBTs in other species of PPNs (*e.g*., *Heterodera schachtii*, *B. xylophilus*, *G. pallida*, and *M. hapla*)^13^ to develop new strategies for their management and control.

In conclusion, we identified the *MiDaf16-like* and *MiSkn1-like* gene families in the genome of *M. incognita* and confirmed its modulation in response to oxidative stress, its expression level in the different life stages of the nematode, and its upregulation during plant parasitism. Next, we showed the efficient *in planta* production of siRNAs, the successful uptake of siRNAs by the nematode, and the downregulation of the *MiDaf16-like1* and *MiSkn1-like1* genes and, consequently, the genes in their networks. Additionally, we observed that single- or double-gene silencing plants of *A. thaliana* or *N. tabacum* showed high resistance to *M. incognita*. In addition, considering the high conservation of these gene families, our data suggest that NBTs can also be developed for modulation of these target genes in other species of PPNs. Finally, our findings showed that these two TF families are powerful targets for the development of NBTs to control and manage nematodes in economically important crops.

## Material and Methods

### *In silico* analysis of *Daf-16* and *Skn-1* TFs from nematodes

All gene sequences of *M. incognita* were retrieved from BioProject ID PRJEB8714 (sample: ERS1696677)^38^ from the online WormBase database version WBPS13^67^. Pairwise identity matrices from nucleotide and amino acid sequences were generated using the Sequence Demarcation Tool Version 1.2 software^68^. Positional conservation of the FOXO (Forkhead box) domain was generated from multiple sequence alignment using Color Align Conservation software^69^. In addition, the conserved domains in gene sequences were checked using the Conserved Domain Database (CDD)^70^. Phylogenetic analyses were performed using the Phylogeny.fr web service^71^. For this, sequences were aligned with MUSCLE software^72^, and the alignment was curated by the Gblocks model. Then, phylogenetic analyses were performed using the maximum likelihood method by PhyML software using the Approximate Likelihood-Ratio test (aLRT) SH-like branch support and GTR and WAG substitution models for nucleotide and amino acid sequences, respectively. Phylogeny trees were generated and visualized by TreeDyn software also implemented in this same web service. In addition, the gene and protein sequences from other nematode species used in these sequence analyses were also retrieved from the WormBase database version WBPS13 database. The expression levels of *MiDaf16-like1*, *MiSkn1-like1*, and some genes from their networks at different stages of the *M. incognita* life cycle were determined using transcriptome datasets (BioProject number: PRJNA390559) retrieved from the BioSample database (NCBI) (Supplemental Table 2). Fifteen transcriptome libraries from *M. incognita* egg, J2, J3, J4, and female stages were generated by Choi *et al*.^39^ using the Truseq RNA Sample Prep Kit (Illumina), and mRNAs were paired-end sequenced (2×101 bp) using Illumina HiSeq. 2000 technology (Supplemental Table 2). The libraries were downloaded and trimmed, and the transcripts were mapped using the genome reference retrieved from the WormBase Parasite database (BioProject PRJNA 340324)^73^. The number of reads mapped in interest target genes was normalized in reads per million (RPM). Additionally, the expression profile of *MiDaf16-like1* to 19 and *MiSkn1-like1* to 4 in different nematode life stages was estimated from these same transcriptome libraries (Supplemental Fig. 1). Features of the *MiDaf16-like* and *MiSkn1-like* genes from *M. incognita*, such as conserved domains in the gene sequences were identified using CDD Database from NCBI^74^, and PFAM Database from EMBL-EBI^75^. NES motifs were predicted using NetNES 1.1 Server^76^, while NLS motifs were predicted using the NLStradamus online tool^77^.

### *Meloidogyne incognita* J2 inoculum

*M. incognita* J2 race 3 and *M. incognita* J2 strain Morelos were obtained from tomato plants (*Solanum lycopersicum cv. Santa Clara*) inoculated and maintained for eight weeks under greenhouse conditions. Infected roots were washed and macerated using a blender after treatment with 0.5% sodium hypochlorite. Eggs were harvested, rinsed with tap water and subsequently separated from root debris using 100 to 550-μm sieves^78^. Then, the eggs were hatched under aerobic conditions at 28 °C, and J2 were harvested every two days, decanted and quantified under a microscope using counting chambers.

### *MiDaf16-like1* and *MiSkn1-like1* expression profiles in response to oxidative stress, host plant parasitism, and different nematode life stages

96-well plates containing 40 freshly hatched *M. incognita* J2 in a final volume of 100 µl were exposed in a gradient ranging from 0 to 5 mM H_2_O_2_ as described by Vicente *et al*.^61^. After overnight exposure to H_2_O_2_ conditions, the nematode survival rate was evaluated under a stereomicroscope. Six biological replicates were performed per treatment. Nematodes were considered dead if no movements were observed after mechanical and light stimulation. Then, tubes containing approximately 5,000 freshly hatched J2 in a final volume of 1 ml were exposed to 0.1, 0.4 and 0.8 mM H_2_O_2_ and incubated in the dark without agitation at 28 °C for 4 and 12 hours. The J2 were harvested, and the total RNA was isolated using TRIzol Reagent (Invitrogen, Carlsbad, CA, USA), mild sonication and TissueLyser II (Qiagen, Hilden, Germany). The RNA concentration was estimated using a spectrophotometer (NanoDrop 2000, Thermo Scientific, Massachusetts, USA), and integrity was evaluated with 1% agarose gel electrophoresis. RNA samples were treated with RNase-free RQ1 DNase I (Promega, Madison, Wisconsin, USA) according to the manufacturer’s instructions. Then, 2 μg of DNase-treated RNA was used as a template for cDNA synthesis using Oligo-(dT)_20_ primer and SuperScript III RT (Life Technologies, Carlsbad, CA, USA), according to the manufacturer’s instructions. The cDNA was quantified by spectrophotometry and diluted with nuclease-free water to 200 ng/µl. RT-qPCR assays were performed in an Applied Biosystems 7500 Fast Real-Time PCR System (Applied Biosystems, Foster City, CA, USA) using 400 ng of cDNA, 0.2 µM of each gene-specific primer (Supplemental Table 3) and GoTaq^®^ qPCR Master Mix (Promega, Madison, Wisconsin, USA). The conditions for qPCR included an initial 95 °C for 10 min, then 40 cycles of 95 °C for 15 s and 59 °C for 50 s, followed by a final melting curve analysis. The expression of the *MiDaf16-like1* (Minc3s02528g30466) and *MiSkn1-like1* (Minc3s02028g27861) genes was normalized using the *Mi18S* (GenBank accession U81578)^79^ and *MiACT* (Minc3s00730g16611) endogenous reference genes in *A. thaliana* and *N. tabacum*, respectively. In addition, *MiSod-3like1*, *MiGst1-like1*, and *MiTTL-5-like1* gene expression were also evaluated after J2 exposure to H_2_O_2_ and normalized using the *Mi18S* gene. Newly hatched J2 in Milli-Q water were used as a control for relative expression levels for both J2 exposed to H_2_O_2_ and J2 during plant parasitism. All samples were carried out in technical triplicate reactions. Primer efficiencies and target-specific amplification were confirmed by a single and distinct peak in the melting curve analysis. The relative expression level (fold change) was calculated using the 2^−∆Ct^ or 2^−∆∆Ct^ method^80^. The *MiDaf16-like1* and *MiSkn1-like1* gene expression in different life stages of *M. incognita* was determined from the egg mass harvested from tomato roots, J2 newly hatched in Milli-Q water at 28°C, J3 harvested from potential galls (stained with fuchsin) of tobacco at 12 to 15 dpi, J4 harvested from potential galls (stained with fuchsin) of tobacco at 20 to 24 dpi, and females harvested from galls of tobacco at 35 to 40 dpi. The total RNA and cDNA were prepared as described above, while the relative expression level (fold change) was normalized with the *β-tubulin* (*MiTUB*) and *glyceraldehyde 3-phosphate dehydrogenase* (*MiGAPDH*) endogenous reference genes.

### Binary vectors and agrobacterium-mediated plant transformation

Three binary vectors were synthesized by the company Epoch Life Science (Sugar Land, TX, EUA) and subsequently transformed into the *A. tumefaciens* strain GV3101. The first binary vector was designed to negatively regulate the *MiDaf16-like1* gene. Thus, two short fragments comprising the regions 578 to 676 and 2002 to 2108 from the *MiDaf16-like1* gene were cloned *in tandem*, in both the sense and antisense orientations and separated by the PDK intron (Fig. 4A, and Supplemental File 1). The second binary vector was designed to negatively regulate the *MiSkn1-like1* gene. Thus, one short fragment comprising the region 2283 to 2624 from the *MiSkn1-like1* gene was cloned in sense and antisense and was also separated by the PDK intron. The third binary vector was designed to negatively regulate the *MiDaf16-like1* and *MiSkn1-like1* genes, simultaneously. For this, these same DNA fragments described above were also cloned into the double-gene silencing vector (Fig. 4A, and Supplemental File 2). It is expected that the siRNAs produced from the transgenes will target the highly conserved regions comprising the FOXO and bZIP domains, respectively (Supplemental Files 1 and 2).

*Arabidopsis thaliana* ecotype Col-0 was genetically transformed by the floral dip method^81^, while *N. tabacum* var. SR1 Petite Havana was transformed from young leaves of 4–5 weeks old, according to Park *et al*.^82^. *N. tabacum* plants were selected *in vitro* using 5 mg/L glufosinate-ammonium (FINALE, Liberty Link, Bayer). Both *A. thaliana* and *N. tabacum* were screened *in vivo* by glufosinate ammonium spraying, confirmed by conventional PCR using specific primers (Supplemental Table 3) and a quick test strip kit (QuickStix™ Kit for PAT/bar, EnviroLogix, Inc., USA) according to the manufacturer’s instructions. Several independent transformants were chosen for propagation, and several T_2_ lines of each construct were chosen for the bioassays.

### Evaluation of resistance of the transgenic plants to *M. incognita*

The *A. thaliana* seedlings were transplanted to pots containing 45 g of autoclaved sand:substrate mixture (1:1; w-w) and maintained in a growth chamber with a 12 h photoperiod and 22 ± 2 °C temperature. Two weeks after transplanting, plants were inoculated with 500* M. incognita* J2 race 3 or 200 J2 strain Morelos (suspended in distilled water). Fifteen plants per line were used, and the experiment was repeated at least two times, while WT plants were used as susceptible controls. At 60 days post-inoculation (dpi), the plants were evaluated for the number of eggs per gram of root, number of J2 hatched per gram of root, number of galls per plant, number of egg masses per plant, and NRF. The *M. incognita* NRF in transgenic plants was determined using Oostenbrink’s formula: NRF = final J2 number/initial J2 number or nematode final population/initial population^83,84^. In contrast, *N. tabacum* seedlings from T_2_ lines were transplanted to pots containing 125 g of sterile sand:soil mixture (1:1; w-w) and maintained in the greenhouse conditions. Eight days after transplanting, plants were inoculated with 1,000* M. incognita* J2 (suspended in distilled water). Sixteen plants per transgenic line were used, while WT plants were used as susceptible controls. At 60 dpi, the plants were evaluated for the number of eggs per gram of root, number of J2 hatched per gram of root, number of galls per plant, and NRF. For morphological analysis, galls were harvested from nematode-infected roots of *A. thaliana* WT, single- or double-gene silencing plants at 45 dpi. Then, they were fixed in 2% glutaraldehyde in 50 mM PIPES buffer, pH 6.9, subsequently dehydrated and embedded in Technovit 7100 (Heraeus Kulzer, Wehrheim, Germany) as described by the manufacturer. Nematode-induced galls were sectioned (3 µm), stained in 0.05% toluidine blue and mounted in Depex (Sigma-Aldrich, St Louis, Missouri, USA). Microscopy analyses were carried out using bright-field optics, and images were acquired with a digital camera (Axiocam, Zeiss, Oberkochen, Germany).

### Expression levels of *MiDaf16-like1*, *MiSkn1-like1*, and genes from their networks during *M. incognita* infection in transgenic plants

Galls from five *A. thaliana* plants (from WT and transgenic plants) of each line were harvested at 60 days post-inoculation and processed in pool form. Then, samples were ground in liquid nitrogen using a mortar and pestle and stored at −80 °C. RNA total was isolated using Concert Plant RNA Purification (Invitrogen, Carlsbad, CA, USA) plus PVP-40 according to the manufacturer’s specifications. Highly pure RNA was used to estimate the relative expression levels of the *MiDaf16-like1* and *MiSkn1-like1* genes during infection of transgenic plants, as described above. RNA isolated from galls harvested from WT *A. thaliana* was used as a positive control of the expression of the gene of interest. In addition, from these same RNA samples, we also validated the consequent downregulation of some network genes of the DAF-16 and SKN-1 TFs involved in nematode defense against stresses. The *MiPRDX2-like1*, *MiSod3-like1*, *MiGPX-like1*, *MiGst1-like1*, and *MiSod1-like1* genes were evaluated in *M. incognita* harvested from four single- or double-gene silencing plants. The expression level was represented as the fold change calculated with the 2^-^^∆CT^ formula using the *glyceraldehyde 3-phosphate dehydrogenase* (*MiGAPDH*) gene as the endogenous reference gene (Supplemental Table 3).

## Supplementary information

Supplementary Information.
