# Supplementary material for: MiDaf16-like and MiSkn1-like gene families are reliable targets to develop biotechnological tools for the control and management of Meloidogyne incognita
Source: Sci Rep. 2020 Apr 24;10:6991. doi: 10.1038/s41598-020-63968-8 (PMC7181638; doi:10.1038/s41598-020-63968-8)
Supplement: Supplementary file 1 — Supplementary Information. [file 41598_2020_63968_MOESM1_ESM.docx]

***MiDaf16*-*like* and *MiSkn1-like* gene families are reliable targets to develop biotechnological tools for the control and management of *Meloidogyne incognita***

Marcos Fernando Basso^1§^, Isabela Tristan Lourenço-Tessutti^1^, Reneida Aparecida Godinho Mendes^1,2^, Clidia Eduarda Moreira Pinto^1,2^, Caroline Bournaud^3^, François-Xavier Gillet^3^, Roberto Coiti Togawa^1^, Leonardo Lima Pepino de Macedo^1^, Janice de Almeida Engler^4^, and Maria Fatima Grossi-de-Sa^1,5§^

^1^ Embrapa Genetic Resources and Biotechnology, Brasília-DF, 70297-400, Brazil;

^2^ Federal University of Brasília, Brasília-DF, 70910-900, Brazil;

^3^ Université de Grenoble Alpes, CNRS, CEA, INRA, 38054 Grenoble Cedex 9, France;

^4^ UMR Institut Sophia Agrobiotech INRA/CNRS/UNS, Sophia Antipolis, France;

^5^ Catholic University of Brasília, Brasília-DF, 71966-700, Brazil;

**^§^ Corresponding author** ([marcosbiotec@gmail.com](mailto:marcosbiotec@gmail.com); [fatima.grossi@embrapa.br](mailto:fatima.grossi@embrapa.br))

Embrapa Genetic Resources and Biotechnology, PqEB Final, W5 Norte, PO Box 02372, 70770-901, Brasília-DF, Brazil. Phone: +55 (61) 99965 6736.

**Supplemental Table 1:** Features of the *MiDAF16-like* and *MiSkn1-like* genes from *Meloidogyne incognita*. Gene sequences were retrieved from BioProject ID PRJEB8714 (sample: ERS1696677)^38^ from the WormBase database version WBPS13 ^67^. Conserved domains in the gene sequences were identified using CDD Database from NCBI ^81^, and PFAM Database from EMBL-EBI ^82^. NES motifs were predicted using NetNES 1.1 Server ^83^, while NLS motifs were predicted using the NLStradamus online tool ^84^.

| **Orthologue** | **Gene ID** | **Nucleotide length** | **Protein length** | **Conserved domain (CDD)** | **Conserved domain (PFAM)** | **NES motif** | **NLS** |
| --- | --- | --- | --- | --- | --- | --- | --- |
| *MiDaf16-like1* | Minc3s02528g30466 | 2,367 | 788 | Cdd:smart00339 | PF00250.18 | yes | yes |
| *MiDaf16-like2* | Minc3s00293g09565 | 2,663 | 853 | Cdd:cd00059 | PF00250.18 | yes | yes |
| *MiDaf16-like3* | Minc3s06738g40249 | 1,629 | 542 | Cdd:cd00059 and Cdd:COG5025 | PF00250.18 | yes | yes |
| *MiDaf16-like4* | Minc3s02143g28529 | 1,194 | 388 | Cdd:cd00059 | PF00250.18 | yes | no |
| *MiDaf16-like5* | Minc3s03756g34708 | 1,527 | 381 | Cdd:cd00059 | PF00250.18 | yes | no |
| *MiDaf16-like6* | Minc3s06700g40200 | 726 | 241 | Cdd:smart00339 | PF00250.18 | yes | no |
| *MiDaf16-like7* | Minc3s00670g15892 | 902 | 284 | Cdd:smart00339 | PF00250.18 | yes | no |
| *MiDaf16-like8* | Minc3s00896g18634 | 1,256 | 310 | Cdd:smart00339 | PF00250.18 | yes | no |
| *MiDaf16-like9* | Minc3s05371g38122 | 813 | 212 | Cdd:pfam00250 | PF00250.18 | yes | no |
| *MiDaf16-like10* | Minc3s01745g26020 | 699 | 232 | Cdd:pfam00250 | PF00250.18 | yes | no |
| *MiDaf16-like11* | Minc3s01171g21384 | 1,164 | 387 | Cdd:pfam00250 | PF00250.18 | yes | yes |
| *MiDaf16-like12* | Minc3s00913g18806 | 3,066 | 794 | Cdd:cd00059 and Cdd:COG5025 | PF00250.18 | yes | yes |
| *MiDaf16-like13* | Minc3s00459g12679 | 1,693 | 510 | Cdd:pfam00250 | PF00250.18 | yes | no |
| *MiDaf16-like14* | Minc3s09607g43370 | 1,402 | 446 | Cdd:pfam00250 | PF00250.18 | yes | no |
| *MiDaf16-like15* | Minc3s03624g34358 | 923 | 278 | Cdd:smart00339 | PF00250.18 | yes | no |
| *MiDaf16-like16* | Minc3s00600g14903 | 1,705 | 511 | Cdd:pfam00250 | PF00250.18 | yes | no |
| *MiDaf16-like17* | Minc3s01319g22739 | 1,318 | 432 | Cdd:pfam00250 | PF00250.18 | yes | no |
| *MiDaf16-like18* | Minc3s00100g04542 | 1,698 | 389 | pfam00250 and Cdd:COG5025 | PF00250.18 | yes | no |
| *MiDaf16-like19* | Minc3s02176g28694 | 3,075 | 919 | Cdd:pfam00250 and Cdd:pfam16159 | PF00250.18 and PF16159.5 | yes | no |
| *Daf-16 - C. elegans* | R13H8.1c | 1,533 | 510 | Cdd:cd00059 | PF00250.18 | yes | yes |
| *MiSkn1-like1* | Minc3s02028g27861 | 1,121 | 345 | Cdd:pfam03131 | no hits | - | yes |
| *MiSkn1-like2* | Minc3s02028g27862 | 1,338 | 369 | Cdd:pfam03131 | PF03131.17 | - | yes |
| *MiSkn1-like3* | Minc3s08604g42418 | 420 | 140 | Cdd:pfam03131 | PF03131.17 | - | yes |
| *MiSkn1-like4* | Minc3s03116g32841 | 1,106 | 227 | Cdd:cd14720 | PF03131.17 | - | yes |
| *Skn-1 - C. elegans* | CCD62212 | 1,872 | 623 | Cdd:cd14698 | PF03131.17 | - | yes |

**Supplemental Table 2:** Transcriptome datasets from *Meloidogyne incognita* at different stages of its development retrieved from the BioSample database (NCBI). The nematodes were maintained in tomato plants (*Solanum lycopersicum* var. Rutgers) under greenhouse conditions (25°C, 16/8 h day/night period) in South Korea ^39^.

|  | **Transcriptome of *Meloidogyne incognita* stages** | | | | |
| --- | --- | --- | --- | --- | --- |
| **Information** | **Egg** | **J2** | **J3** | **J4** | **Female** |
| Biosample number | SAMN07174878 | SAMN07174881 | SAMN07174884 | SAMN07174965 | SAMN07174968 |
| Experiment | SRX2919283 | SRX2919273 | SRX2919274 | SRX2919285 | SRX2919279 |
| Library name | MI-egg_1 | MI-J2_1 | MI-J3_1 | MI-J4_1 | MI-Female_1 |
| Sequences per library | 75,927,670 | 71,481,164 | 59,115,050 | 68,172,904 | 81,833,702 |
| Reads mapped in *MiDaf16-like1* | 2,128 | 843 | 1,027 | 884 | 1,184 |
| Reads mapped in *MiSkn1-like1* | 294 | 346 | 1,250 | 392 | 408 |
| Biosample number | SAMN07174879 | SAMN07174882 | SAMN07174885 | SAMN07174966 | SAMN07174969 |
| Experiment | SRX2919286 | SRX2919275 | SRX2919278 | SRX2919281 | SRX2919280 |
| Library name | MI-egg_2 | MI-J2_2 | MI-J3_2 | MI-J4_2 | MI-Female_2 |
| Sequences per library | 67,663,576 | 56,144,924 | 47,651,268 | 53,077,044 | 56,174,662 |
| Reads mapped in *MiDaf16-like1* | 1,892 | 663 | 829 | 700 | 920 |
| Reads mapped in *MiSkn1-like1* | 226 | 261 | 977 | 340 | 328 |
| Biosample number | SAMN07174880 | SAMN07174883 | SAMN07174886 | SAMN07174967 | SAMN07174970 |
| Experiment | SRX2919272 | SRX2919276 | SRX2919284 | SRX2919282 | SRX2919277 |
| Library name | MI-egg_3 | MI-J2_3 | MI-J3_3 | MI-J4_3 | MI-Female_3 |
| Sequences per library | 67,341,584 | 55,826,392 | 47,260,062 | 52,800,896 | 55,847,170 |
| Reads mapped in *MiDaf16-like1* | 1,872 | 656 | 767 | 693 | 829 |
| Reads mapped in *MiSkn1-like1* | 235 | 252 | 957 | 325 | 338 |

Eggs samples were collected from egg masses and purified by sucrose gradient centrifugation. J2 samples were obtained by hatching a portion of the collected eggs, while J3, J4, and female were collected from infected roots around two and six weeks after infection. Libraries were prepared using Truseq RNA Sample Prep Kit (Illumina), and mRNAs were paired-end sequenced (2x101 bp) using Illumina HiSeq 2000 technology. SRA number: SRP109232; BioProject number: PRJNA390559.

**Supplemental Table 3.** Primers sequence for conventional and real-time PCR assays from transgenic plants and *Meloidogyne incognita*.

| **Gene ID** | **Gene name** | **Assay** | **Primer name** | **Primer sequence (5’-3’)** | ***Tm*** | **GC%** | **Length (bp)** |
| --- | --- | --- | --- | --- | --- | --- | --- |
| Minc3s02528g30466 (Minc3s00913g18806, Minc3s03624g34359) | *MiDaf16-like1* | qPCR | MiDAF16q1(F) | CATGGACATTCTGCTCCCCTT | 59 | 52 | 102 |
|  |  |  | MiDAF16q1(R) | GGCTGTTGTTGCTGCCAAAT | 59 | 57 |  |
| Minc3s02028g27861 (Minc3s03384g33686, Minc3s03613g34320) | *MiSkn1-like1* | qPCR | MiSKN1q1(F) | TCCAACCACCAACAGCAACA | 59 | 50 | 84 |
|  |  |  | MiSKN1q1(R) | ACGTGAACGTTGCCTTGAATG | 59 | 47 |  |
| Minc3s00535g13905  (Minc3s00365g11069,  Minc3s06441g39819) | *MiPRDX2-like1* | qPCR | MiPRDX2q(F) | TGTTTCTTTGTCTGACTACAAGGG | 55 | 42 | 199 |
|  |  |  | MiPRDX2q(R) | CTCCTTACGTGGCTTATTGATCC | 55 | 48 |  |
| Minc3s03341g33565 (Minc3s03341g33566 Minc3s01794g26348) | *MiSod3-like1*^+^ | qPCR | MiSOD3q(F) | TTTGGAAGTTGCTTGTTGTCC | 58 | 43 | 195 |
|  |  |  | MiSOD3q(R) | TCAGTCTGAGCACTTTCATACC | 58 | 45 |  |
| Minc3s00235g08236 | *MiGPX-like1* | qPCR | MiGPXq(F) | AGATGGGAGATGTTTACAAGGG | 55 | 45 | 169 |
|  |  |  | MiGPXq(R) | GGCTCTTGACTATTAAACTGATTGC | 55 | 40 |  |
| Minc3s00369g11129 | *MiGst1-like1^X^* | qPCR | MiGST1q(F) | AGGAAATGTGGAGCAACTTCG | 56 | 47 | 161 |
|  |  |  | MiGST1q(R) | TCATCATTCCCGTAAAGTGAGC | 56 | 45 |  |
| Minc3s08143g41941 | *MiSod1-like1*^+^ | qPCR | MiSOD1q(F) | TACTGAACTCCGAGCTATTTGC | 55 | 45 | 162 |
|  |  |  | MiSOD1q(R) | CCATAAGCATGAACATGGAAACC | 55 | 43 |  |
| Minc3s01149g21190, Minc3s03007g32403 | *MiTTL5-like1^Y^* | qPCR | MiTTL5q(F) | CCATTAGCGGAAGATTTATTTGTGG | 55 | 40 | 198 |
|  |  |  | MiTTL5q(R) | CGTGTCCAAACATAAAGAACGG | 55 | 45 |  |
| Transgene | *bar/PAT* | PCR | bar/PAT | GACGGGGCGGTACCGGCAGG | 57 | 80 | 400 |
|  |  |  | bar/PAT | CCGCAGGAGTGGACGGACGAC | 57 | 71 |  |
| Minc3s08501g42315 | *Mi18S^*^* | qPCR | Mi18Sq(F) | CTGTGATGCCCTTAGATGTCC | 59 | 52 | 170 |
|  |  |  | Mi18Sq(R) | TGATGACTCGCACTTACTTGG | 59 | 52 |  |
| Minc3s00730g16611 | *MiACT^#^* | qPCR | MiACT(F) | GATTCGTATGTGGGAGATGAGG | 55 | 50 | 194 |
|  |  |  | MiACT(R) | TTAGCCTTTGGGTTGAGAGG | 55 | 50 |  |
| Minc3s00001g00047 | *MiTUB* | qPCR | MiTUB(F) | TGGAAAGTATGTCCCAAGAGC | 60 | 47 | 257 |
|  |  |  | MiTUB(R) | CACCACCAAGCGAGTGAGT | 60 | 58 |  |
| Minc3s07075g40689 | *MiGAPDH* | qPCR | MiGAPDH(F) | GCTTCCTGCACTACTAATTGTCTTG | 60 | 44 | 100 |
|  |  |  | MiGAPDH(R) | CAGTAACAGCGTGTACAGTAGTCAT | 60 | 44 |  |

^*^ Endogenous reference gene from *Meloidogyne incognita*, both J2 and infecting *Arabidopsis thaliana.* ^#^ Endogenous reference gene from *Meloidogyne incognita*, both J2 and infecting *Nicotiana tabacum*. ^+^ *SOD-3*, *SOD-1*, and *PRDX-2* genes act in the nematode antioxidant pathway, which is upregulated by DAF-16 and SKN-1 transcription factors. ^X^ *GST-1* and *GPX* genes act in the nematode detoxification pathway, which is upregulated by DAF-16 and probably SKN-1 transcription factors. ^Y^ *TTL-5* gene coding to effector secreted in response to the oxidative stress response, which interacts with ferredoxin:thioredoxin reductase catalytic subunit from host plant during early stages of nematode infection. This interaction is correlated with ROS scavenging and host susceptibility ^23^.

**Supplemental File 1:** DNA fragment from *MiDaf16-like1* cloned into binary vectors (in red) and the region comprising the FOXO domain.

>Minc3s02528g30466_*MiDaf16-like1*...cloned fragment corresponding to 578 to 676 and 2002 to 2108.

ATGGATTCAGCCAGTCAATTACTTTTATCAGCTTCCCCTTTACAATTACATGCTGCCATTTCTGTTGCTGCAGCACAGGCAGCAGCAGCTGAAGAACAACAACAACAATCTTCTTTGGCAACAGCAGCAGCTACAGCAAAAAATACAGAAATTAAAATTGAAGAACCTGCTGGGGAAGGAAATGGGGGAGAGGAAGCGGCAATGCCTAATTCTTCTGCTGCTCAGAGTCCCCGTCAAACCAGCAATGCTCTCGAAGCAGCTTTTAATCTTAATTTTTTGTTAAAAATGGAAACAGATTCTGATGATAAACTTGCACCTTTGGAGATTAGTGAAAGTATGTCTTCATTGAATGGTTCTTTGCAGACAAGTCCCACAATAATTACTTGCAAAACCCCTCCAGCAAATGGAAAGTCAATGTTGCAAGTTATTAAGCCTCCTGGAGATGAAAAGGAAGAAAGGCCTTCATTAAGTTACAAAGATTTAATTATCGAAGCTATTGAAAGTAATCCGGATAGAAGATTAAAATTAAGTGAAATCTACCAAGTAATTAAATATTTACATCCGTATTATCAAAGAAGAGCCGATCAATGGGGATGGCAAAATTCTATTAGACATAATCTTTCTTTACATGATTGTTTTGTTAAATTGCCATTAAAACAGACTAGTGCTAATGGGGTTGTTGGTCACTTCTGGACTGTAGTTCATCGTAACCCTGAAGATAAACAGGGGCCAACAAGAAGACGTTCTCGTGCAAATGGCACAGGCGGTGGACCTACTGGAAAAGGGTCTAGAAAAGTAGGAGGGAATCAAGGTCACCCAGCATCTCAAGTTGGAAATTCTGCTGGAGGGAAGGCAAAAGGATTGAAAGGAAGGCAAAGTGTTAGCCTTTCCCACCTCTTCAGCTCTGACAGCGGTGTTATGAGTGACGATTGTGGTGGTGGTACACAATCTGGAGATATTAGTCCAACTAATGGTAGCGCCTCAGTTCCGGCTTCTTTACTGCAAATTAATACCTCTTCTGATTTATTGACAAACAAATTTCAACCTCTTCAATCCCCTCTGGCTTCAAATAGCCTTCAACATTCAGCATTACAATTAGCGTTAAAACAACAAGCTGCTGCTGCTCTTAATTTTAATAATAATGGAATGAATTTGAATCAGAATAATCTTCTTTCCCCTCTCGAGCTTGTTTTGGGAAGTCAAGCTGTAGCTCAGCATTTACTTGCTGCCCAAACACCTCAACATCACCACCAACAACAACAATTATTACAATTTTCTCCAATTCCTGGCAAAACAGCCCCTTCACAACCAGCATTATTAGAACAACATTTACAGCAATTATTAGCTATCGCTGCTGGAGCACAACAAGATCAACTACTAACTCAGCCAGAAACACCTGCATCTGCTCCTCCTTCTTTACCTGCCAATCCATTAGCACCAACAACTTCTAATTCAACATTAGCTACAACACCAACAACAAATGGAGGAGGTTTAAATCTGCTCAGTCAATTAGCTAGCCAGGCATTAGCTTCTACAAGTCCACAACCTACACAACAACCTCAACAACAACAAGATCAAGCTTCTTTAGCGGTTGCAGCAGCTGCTGCAGCAGAATTACAACGTATTCAGTTGGTTCAGTTATATGCACAACAACAACAGTTACAACAACAATTAGCCCTTTTGGAGCACACCCACAGAGAACATTTAAATCTTCGTTTACTTGGTGGACATCGTCATAATGATTTTCCTCATGGACATTCTGCTCCCCTTAGTCCAGTTGTTCACGATCCATTAACAACAACTTTAAATCGAAATAATCCTCTACTTCTTAATTTGGCAGCAACAACAGCCCCTTCTTCTCCATCAGTTTTGGCAAATGAAAATGGAAGGAATAATTCAAATAATAATCAATCAATGTTAACACAATTATTATTATCAAGATTATTTTCTTCTGCTGCTTCTACAAATAATAATGAAAATAATTCAACAAATGTTGAACAACAGCCAACAACTAATTTACCTGACAATTCCCCACCCCCTCCATCATCTGTCCCCGCTGTTGCACACTCTCCTGTTTCTGAAAGTGTTAATCAACAACAAGTTGAATTACAACAAGCAGCTCTATTGCTTGAACGTTATCAACAGCAACAACAACAACTTTTGTTAATTGACCAATTCCAACAACAACAGCAACAATTAGCAGAAGTCGGACAACTGCAAGCGCTTACTCAAGTATTTGGTACTGCAGCAGCTGCTGCTGCCGCGGCTGCCGCTGTTGTTGAACAAGTCCAAGAGGCACAACAACAAATTGCTGAGCAACAAATTGTTGAACAACAAGAAAGTGAGGAAGTTGTTTCTGTTGTTCATTTAAATATTGTTAATTTTCTGTTTGTATTTGTTTGA

FOXO domain and RNAi target

RNAi target

**Supplemental File 2:** DNA fragment from *MiSkn1-like1* cloned into binary vectors (in red) and the region comprising the bZIP domain.

>Minc3s02028g27861_*MiSkn1-like1*...cloned fragment corresponding to 2283 to 2624.

ATGCCGTCTTCAAACGATGCTGAGGAGCCTGGGGAGACAACAGCTATAATTTCTACTCATCAATTCCAGCCTCCACAAATTTTACATCTTATTTCTAATACTGAAAATGCTTCATTCCATCCAAGACGACGTCGAATTACTAGAAGATCTGCCTCTAGAATGGATTCCCGTGAAAGATTTTTGGCTATGAAAGGGCGCAGTTCATATTCTAAGGCTGGAAGTCAGCAAGCAAGCATGTCGAGTGCAGGTCTGGCGGTTTCTTTATTTCTTTTGGCTGCAATTTGTGGTGCACATTTACTTCGGACAACAACAACAGACCTCACAGATGTATTTAAAAAATCTTCTTTCTCTTCTGATATTCAAATACAATTCATCAAATCTCTTTATTCAAATATTCATCATCATCAACCATTCACTTCTCAACAACTCTATAATTTCGATATTCAAACAAAAATTTCGCAAAATATAAATGAGAGGTTTCAAAGATTTGTAGAGCGTTTGGAAGAATATGTTCCTTCTACGATTACTTCAGCTTCACTAAATGACTGGATGCTTTTCGGACATCAAAGGCAAGAATTACAGGTTAATGTTCTTCTGGCTGAAAGACGTCGTAGAACGGTCCAACGCCAACGCAGTCCAACACCAATTCCTCAATCAATTTCTCCATCTCGATTTATTAATCGACAACGTAATACTTCTTCAGCTCCACCAGATTCTCTTAGAAGTTGGGCTCATTTGTCTACTTCCCCAACATCGATACAACCATCTACTTCTGATTTTGGGACAAATTCTAATCAAACTGATGAACCAACACTTATGGATTATGATGCTATTGATTCTTATTGGAGATGGGACATTGACCAAGAAAAGGGAGGATTATCGCCCTATCAAAGATTAAATGATTTTGGTCAGCAATTAGATGAAGATTATTATTATTTAAATAATGAAAGAAATTTTCAAGAGGCAGATATTCTTTTTGAGCGTGATATTCAATTGCTTACAGAGAAGGGTTTATTGCCAGACCAGACTAATTTTTTTCAAGACAATTTAAATGACAATGAGGTTAATCTTTTGGCTAATTTCTCTAGAACTCTCAGTTTTGATGGAATTGTTGAAAATCAGAATGAAAATACAAAATGGAATGATTTATTACAATTACCTACAAATAGAAAAACATCAATTATTTCTCAATCTTCTTCATATAATCCTCAGATTGACGAATCAGAATTGCCCAATATTCCGATTTATTCTCCTTTTCAAACAGAAAATAATCAAAATTTAAATCAAAAGGATTCTGTTCCAAATCAAAGAGAAGTAGGCTGTTCATCCAGAGAATCTATTTCAGCATTATCTGATATTAGACAACCTATTTTATTTAATGATGTTTCATTGGCCCGTTGTTCTTCCTCATCGACTTCAATTGGGGAAAATAGCGATAATGAAGAGATGGTAATGGAACGGCAACAAATGCTTGAAGAACAATCTTTGCAACATTCTATTACTAATCAACAACAATCTATTCCATTAGCTGATCATTTTTCTGAAAAAGTATTAACCGAAAAGAATCAAAAAACTGAAAAAGACGAGGAAGCAAAGGACGAAGCTGAAACAGAAATGCTTTTATCAGCACTTTTTCCTCAATTATTTTCACCTCTCCCAATTAATGATCCAAGGTCGTCCTCACAACAAAATTCTCCATTATTTTCTGATGGAAATTTAGTGAACAATAGTGAAGTTGAAGCAATTCTTCATGAGTTGGCTGCAGCAGAGGTTATTTTAATGAACAGTACCCAAACGTTGGCCCAAAACAATTTTTTATCAAATCAACTACAAACTTCAATTGAACAAATTGCAAACACCCAACAACAACCTTCATCATTATCACCTTTAGCTTTACCCATTTCACCTTCAATTGGGCCTCTTTCACCCATTTTTGAACATTTTAATCAAACTTTAAATATGAATGGTTCTCCTCCTTCTCTTCCATTTGACGCTAATGGGAATTCTTTTAATCTTTTTGGGGCTCCACTAAATCTGTATTGCAACACTACTGTTCAAAATAGTTGTTCCCCTACTACGAGTTCATCCTCTGTCGGGTTACCTGTAACTGATTCTAACATTGTTTCATTCAATTCTCCAACAGAAGTAGTTGGTTCTTCAGAATTATCTAACAGTGATTCTTTTGCCTTCCGTTACGAACAAAAATTAATTCCAAAAATTTCTGATAATTTTCAAAAAATAAAAAACCCGCCAATTAAAAGTTTGGAAGGACAAGTCCCAATGAAAGCTAAAGGAAAAAGAGGGCGAAGATCGAAAGATGATTCCCTTGTAAATCAATACGATCTTCCTTATTCTGCTGAACATCTTACAGCAATGTCTTATCGTGATTATTCTTCCTTAATGCAGGATGTTCGTTTAACTAGTCAACAAAAAGCTTTAATTAAAAAAATTAGAAGAAGAGGTCGAAATAAATTGGCAGCGAGAAAGTGTCGAGACCGTCGTTTAAAGAATGAAGCTAGATTTGATGGGGAAGTGGTTTTTGATGAATATATCGAAGATGAAGAAGATTGGGATGAGGATATTGATGTTGTTGATGTTGATGATTTAAGTATTGTTAATAAATGGAGAAATGTTAGCAAATCAAAATTTAGTAATGAAGGAAATGATTTTGAAAAACAATTTGGAGAAAATAAGTTTAAACAAGAAAGTATTTCTCGTTTTCCGTCTACCTCAGCATATGCAGACAATATTAATTCCAACCACCAACAGCAACAATTTCTTCCTTTAACTCAAAATATTTTAAATGAAAATACTTCTCATTCAAGGCAACGTTCACGTAAACAACAACATCCCTCTACACAACAATTTGCTATTCGGGTTGAAATTTGA

bZIP domain and RNAi target

**Supplemental File 3:** *MiDaf16-like1* gene CDS sequence from_*M. incognita* race 3 (Brazilian isolates) retrieved from data mining of the genome sequencing (Bioproject: PRJNA480412; Experiment: SRX4373676).

ATGGATTCAGCCAGTCAATTACTTTTATCAGCTTCCCCTTTACAATTACATGCTGCCATTTCTGTTGCTGCATCACAGGCAGCAGCAGCTGAAGAACAACAACAACAATCTTCTTTGGCAACAGCAGCAGCTACAGCAAAAAATACAGAAATTAAAATTGAAGAACCTGCTGGGGAAGGAAATGGGGGAGAGGAAGCGGCAATGCCTAATTCTTCTGCTGCCCAGAGTCCCCGTCAAACCAGCAATGCTCTCGAAGCAGCTTTTAATCTTAATTTTTTGTTAAAAATGGAAACAGATTCTGATGATAAACTTGCACCTTTGGAGATTAGTGAAAGTATGTCTTCATTGAATGGTTCTTTGCAGACAAGTCCCACAATAATTACTTGCAAAACCCCTCCAGCAAATGGAAAGTCAATGTTGCAGGTTATTAAGCCTCCTGGAGATGAAAAAGAAGAAAGGCCATCATTAAGTTACAAAGATTTAATTATCGAAGCTATTGAAAGTAATCCAGATAGAAGATTAAAATTAAGTGAAATCTACCAAGTAATTAAATATTTACATCCGTATTATCAAAGAAGAGCCGATCAATGGGGATGGCAAAATTCTATTAGACATAATCTTTCTTTACATGATTGTTTTGTTAAATTGCCATTAAAACAGACTAGTGCTAATGGGGTTGTTGGTCACTTCTGGACTGTAGTTCATCGTAACCCTGAAGATAAACAGGGGCCAACAAGAAGACGTTCTCGTGCAAATGGCACAGGCGGTGGACCTACTGGAAAAGGGTCTAGAAAAGTAGGAGGGAATCAAGGTCACCCAGCATCTCAAGTTGGAAATTCTGCTGGAGGGAAGGCAAAAGGATTGAAAGGAAGGCAAAGTGTTAGCCTTTCCCACCTCTTCAGCTCTGATAGCGGTGTTATGAGTGACGATTGTGGTGGTGGTACACAATCTGGAGATATTAGTCCAACTAATGGTAGCGCCTCAGTTCCAGCTTCTTTACTGCAAATTAATACCTCTTCTGATTTATTGACAAACAAATTTCAACCTCTTCAATCCCCTCTGGCTTCAAATAGCCTTCAACATTCAGCATTACAGTTGGCGTTAAAACAACAAGCTGCTGCTGCTCTTAATTTTAATAATAATGGAATGAATTTGAATCAGAATAATTAGCTTCTTTCCCCTCTTGAACTTGTTTTGGGAAGTCAAGCTGTAGCTCAGCATTTACTTGCTGCCCAAACACCTCAACATCACCACCAACAACAACAATTATTACAATTTTCTCCAATTCCTGGCAAAACAGCCCCTTCACAACCAGCATTACTAGAACAACATTTACAACAATTATTAGCTATTGCCGCTGGAGCACAACAGGATCAATTATTAACTCAACCAGAAACACCTGCATCCGCTCCTCCTTCTTTACCTGCCAATCCATTAGCACCAACAACTTCTAATTCAACATTAGCTACAACACCAACAACAAATGGAGGTTTAAATCTGCTCAGTCAATTAGCTAGCCAGGCATTAGCTTCTACAAGTCCACAACCTTCACAACAACCTCAACAACAAGATCAAGCTTCATTAGCCGTTGCAGCAGCTGCTGCAGCAGAATTACAACGTATTCAGTTGGTTCAGTTATATGCACAGCAACAACAGTTACAACAACAATTAGCCCTTTTGGAGCACACCCACAGAGAACATTTAAATCTTCGTTTACTTGGTGGACATCGCCATAATGATTTTCCTCATGGACATTCTGCTCCCCTTAGTCCAGTTGTTCACGATCCATTAACAACAACTTTAAATCGAAATAATCCTCTACTTCTTAATTTGGCAGCAACAACAGCCCCCTCTTCTCCATCAGTTTTGGCAAATGAAAATGGAAGGAATAATTCAAATAATAATCAATCAATGTTAACACAATTATTATTATCAAGATTATTTTCTTCTGCTGCTTCTACAAATAATAATGAAAATAATTCAACAAATGTTGAACAACAGCCAACAACTAATTTACCTGACAATTCCCCACCCCCTCCATCATCTGTCCCCGCTGTTGCACACTCTCCTGTTTCTGAAAGTGTTAATCAACAACAAGTTGAATTACAACAAGCAGCTCTATTGCTTGAACGTTATCAACAGCAACAACAACAACTTTTGTTAATTGACCAATTCCAACAACAACAGCAACAATTAGCAGAAGTCGGACAACTGCAAGCGCTTACTCAAGTATTTGGTACTGCAGCAGCTGCTGCTGCCGCGGCTGCCGCTGTTGTTGAACAAGTCCAAGAGGCACAACAACAAATTGCTGAGCAACAAATTGTTGAACAACAAGAAAGTGAGGAAGTAGTTTCTGTTGTTCATTTAAATATTGTTAATTTTCTGTTTGTATTTGTTTGA

**Supplemental File 4:** *MiSkn1-like1* gene CDS sequence from_*M. incognita* race 3 (Brazilian isolates) retrieved from data mining of the genome sequencing (Bioproject: PRJNA480412; Experiment: SRX4373676).

ATGCCGTCTTCAAACGATGCTGAGGAGCCTGGGGAGACAACAGCTATAATTTCTACTCATCAATTCCAGCCTCCACAAATTTTACATCTTATTTCTAATACTGAAAATGCTTCATTCCATCCAAGACGACGTCGAATTACTAGAAGATCTGCCTCTAGAATGGATTCCCGTGAAAGATTTTTGGCTATGAAAGGGCGCAGTTCATATTCTAAGGCTGGAAGTCAGCAAGCAAGCATGTCGAGTGCAGGTCTGGCGGTTTCTTTATTTCTTTTGGCTGCAATTTGTGGTGCACATTTACTTCGGACAACAACAACAGACCTCACAGATGTATTTAAAAAATCTTCTTTCTCTTCTGATATTCAAATACAATTCATCAAATCTCTTTATTCAAATATTCATCATCATCAACCATTCACTTCTCAACAACTCTATAATTTCGATATTCAAACAAAAATTTCGCAAAATATAAATGAGAGGTTTCAAAGATTTGTAGAGCGTTTGGAAGAATATGTTCCTTCTACGATTACTTCAGCTTCACTAAATGACTGGATGCTTTTCGGACATCAAAGGCAAGAATTACAGGTTAATGTTCTTCTGGCTGAAAGACGTCGTAGAACGGAATTAAAGTCCAACGCCAACGCAGTCCAACACCAATTCCTCAATCAATTTCTCCATCTCGATTTATTAATCGACAACGTAATACTTCTTCAGCTCCACCAGATTCTCTTAGAAGTTGGGCTCATTTGTCTACTTCCCCAACATCGATACAACCATCTACTTCTGATTTTGGGACAAATTCTAATCAAACTGATGAACCAACACTTATGGATTATGATGCTATTGATTCTTATTGGAGATGGGACATTGACCAAGAAAAGGGAGGATTATCGCCCTATCAAAGATTAAATGATTTTGGTCAGCAATTAGATGAAGATTATTATTATTTAAATAATGAAAGAAATTTTCAAGAGGCAGATATTCTTTTTGAGCGTGATATTCAATTGCTTACAGAGAAGGGTTTATTGCCAGACCAGACTAATTTTTTTCAAGACAATTTAAATGACAATGAGGTTAATCTTTTGGCTAATTTCTCTAGAACTCTCAGTTTTGATGGAATTGTTGAAAATCAGAATGAAAATACAAAATGGAATGATTTATTACAATTACCTACAAATAGAAAAACATCAATTATTTCTCAATCTTCTTCATATAATCCTCAGATTGACGAATCAGAATTGCCCAATATTCCGATTTATTCTCCTTTTCAAACAGAAAATAATCAAAATTTAAATCAAAAGGATTCTGTTCCAAATCAAAGAGAAGTAGGCTGTTCATCCAGAGAATCTATTTCAGCATTATCTGATATTAGACAACCTATTTTATTTAATGATGTTTCATTGGCCCGTTGTTCTTCCTCATCGACTTCAATTGGGGAAAATAGCGATAATGAAGAGATGGTAATGGAACGGCAACAAATGCTTGAAGAACAATCTTTGCAACATTCTATTACTAATCAACAACAATCTATTCCATTAGCTGATCATTTTTCTGAAAAAGTATTAACCGAAAAGAATCAAAAAACTGAAAAAGACGAGGAAGCAAAGGACGAAGCTGAAACAGAAATGCTTTTATCAGCACTTTTTCCTCAATTATTTTCACCTCTCCCAATTAATGATCCAAGGTCGTCCTCACAACAAAATTCTCCATTATTTTCTGATGGAAATTTAGTGAACAATAGTGAAGTTGAAGCAATTCTTCATGAGTTGGCTGCAGCAGAGGTTATTTTAATGAACAGTACCCAAACGTTGGCCCAAAACAATTTTTTATCAAATCAACTACAAACTTCAATTGAACAAATTGCAAACACCCAACAACAACCTTCATCATTATCACCTTTAGCTTTACCCATTTCACCTTCAATTGGGCCTCTTTCACCCATTTTTGAACATTTTAATCAAACTTTAAATATGAATGGTTCTCCTCCTTCTCTTCCATTTGACGCTAATGGGAATTCTTTTAATCTTTTTGGGGCTCCACTAAATCTGTATTGCAACACTACTGTTCAAAATAGTTGTTCCCCTACTACGAGTTCATCCTCTGTCGGGTTACCTGTAACTGATTCTAACATTGTTTCATTCAATTCTCCAACAGAAGTAGTTGGTTCTTCAGAATTATCTAACAGTGATTCTTTTGCCTTCCGTTACGAACAAAAATTAATTCCAAAAATTTCTGATAATTTTCAAAAAATAAAAAACCCGCCAATTAAAAGTTTGGAAGGACAAGTCCCAATGAAAGCTAAAGGAAAAAGAGGGCGAAGATCGAAAGATGATTCCCTTGTAAATCAATACGATCTTCCTTATTCTGCTGAACATCTTACAGCAATGTCTTATCGTGATTATTCTTCCTTAATGCAGGATGTTCGTTTAACTAGTCAACAAAAAGCTTTAATTAAAAAAATTAGAAGAAGAGGTCGAAATAAATTGGCAGCGAGAAAGTGTCGAGACCGTCGTTTAAAGAATGAAGCTAGATTTGATGGGGAAGTGGTTTTTGATGAATATATCGAAGATGAAGAAGATTGGGATGAGGATATTGATGTTGTTGATGTTGATGATTTAAGTATTGTTAATAAATGGAGAAATGTTAGCAAATCAAAATTTAGTAATGAAGGAAATGATTTTGAAAAACAATTTGGAGAAAATAAGTTTAAACAAGAAAGTATTTCTCGTTTTCCGTCTACCTCAGCATATGCAGACAATATTAATTCCAACCACCAACAGCAACAATTTCTTCCTTTAACTCAAAATATTTTAAATGAAAATACTTCTCATTCAAGGCAACGTTCACGTAAACAACAACATCCCTCTACACAACAATTTGCTATTCGGGTTGAAATTTGA


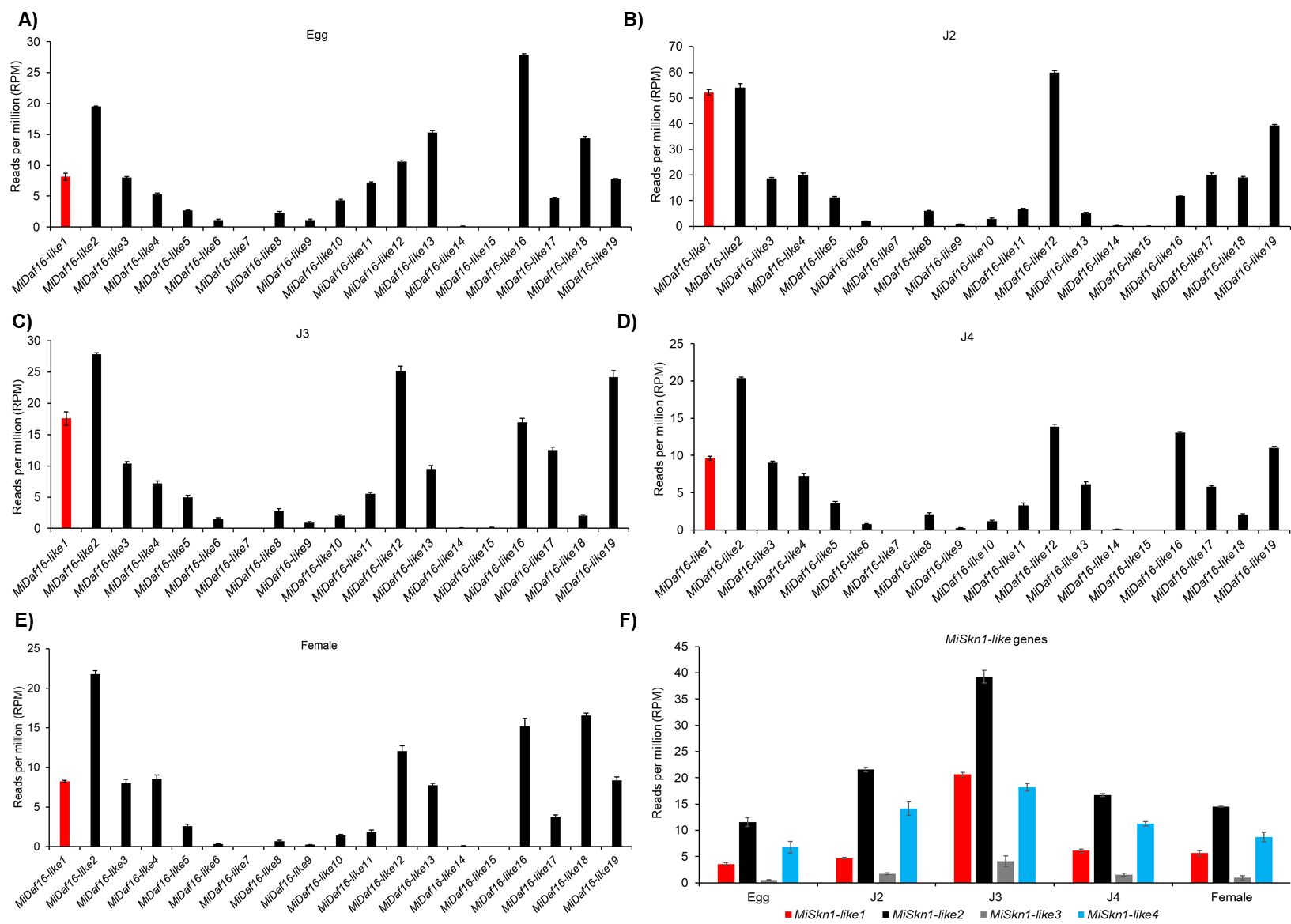


**Supplemental Figure 1.** Expression profile of *MiDaf16-like1* to *19* **(A to E)** and *MiSkn1-like1* to *4* **(F)** in different life stages (egg, J2, J3, J4, and female) of *Meloidogyne incognita* using transcriptome datasets (BioProject number: PRJNA390559; ^39^) retrieved from the BioSample database (NCBI). Error bars represent confidence intervals corresponding to three libraries per life stage of the nematode.


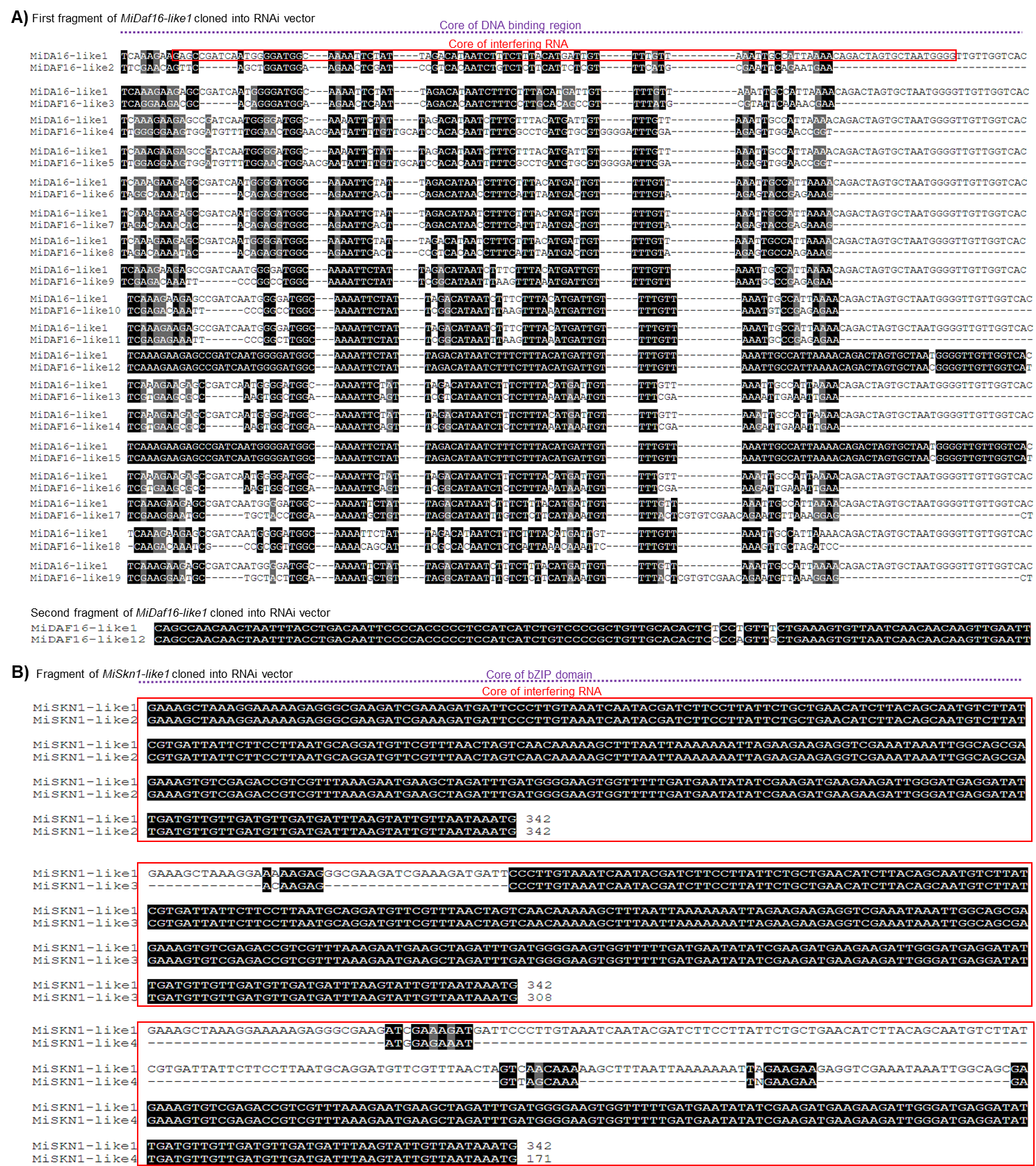


**Supplemental Figure 2.** Sequence alignment to identify regions with high identity. (**A**) Alignment of the *MiDaf16-like1* to *19* sequences for pairwise comparison with *MiDaf16-like1*, showing the gene region cloned in the RNAi vector. These alignments also suggest the potential knockdown of other *MiDaf16-like* genes in addition to *MiDaf16-like1*. (**B**) Alignment of the *MiSkn1-like1* to *4* sequences for pairwise comparison with *MiSkn1-like1*, showing the gene region cloned in the RNAi vector. These alignments also suggest the potential knockdown of three other *MiSkn1-like* genes in addition to *MiSkn1-like1*.


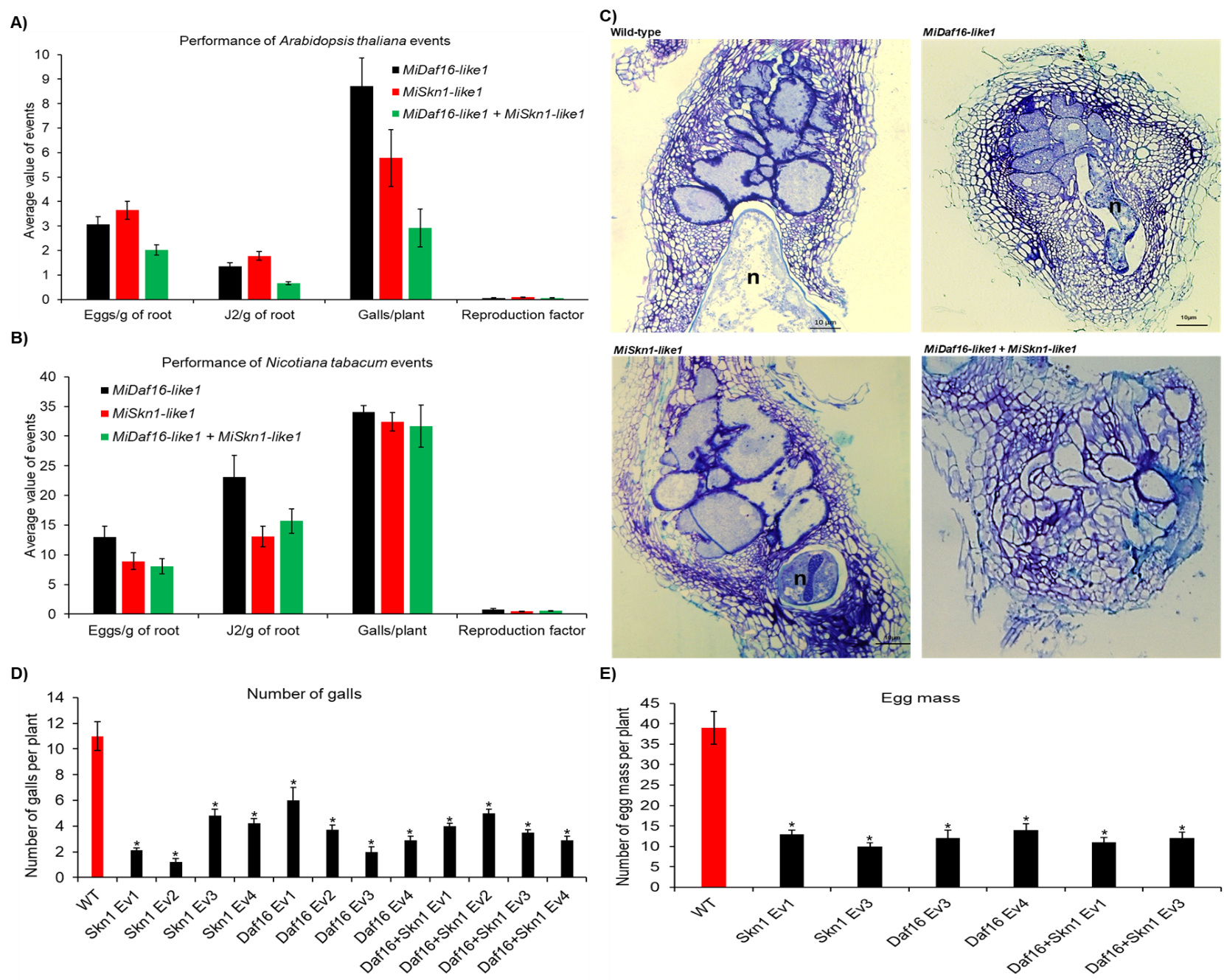


**Supplemental Figure 3.** Performance of the single- and double-gene silencing of the *MiDaf16-like1* and *MiSkn1-like1* genes in *Arabidopsis thaliana* **(A)** and *Nicotiana tabacum* **(B)** plants during infection with *Meloidogyne incognita* J2 race 3. Error bars correspond to the mean of the standard deviation of all lines of each single- and double-gene silencing construct. **(C)** Gall morphology in *Arabidopsis thaliana* roots at 45 days post-inoculation (dpi) with *M. incognita* J2 strain Morelos. The wild-type line showed the phenotype of a typical gall at the end of the nematode life cycle. The *MiDaf16-like1* or *MiSkn1-like1* gene silencing lines showed giant cells lacking the typical dense cytoplasm that were apparently smaller and an apparent delay in nematode development. The *MiDaf16-like1* + *MiSkn1-like1* gene silencing lines showed giant cells lacking the typical dense cytoplasm and were apparently smaller. n: nematode; bars = 50 µm. Evaluation of the resistance level of *A. thaliana* T_2_ plants to *M. incognita* strain Morelos infection. The number of galls per plant **(D)** and the number of egg masses per plant **(E)** were measured at 45 dpi. Error bars represent confidence intervals corresponding to 12 biological replicates (n=12). Asterisks indicate significant differences based on Tukey’s test at 5%.


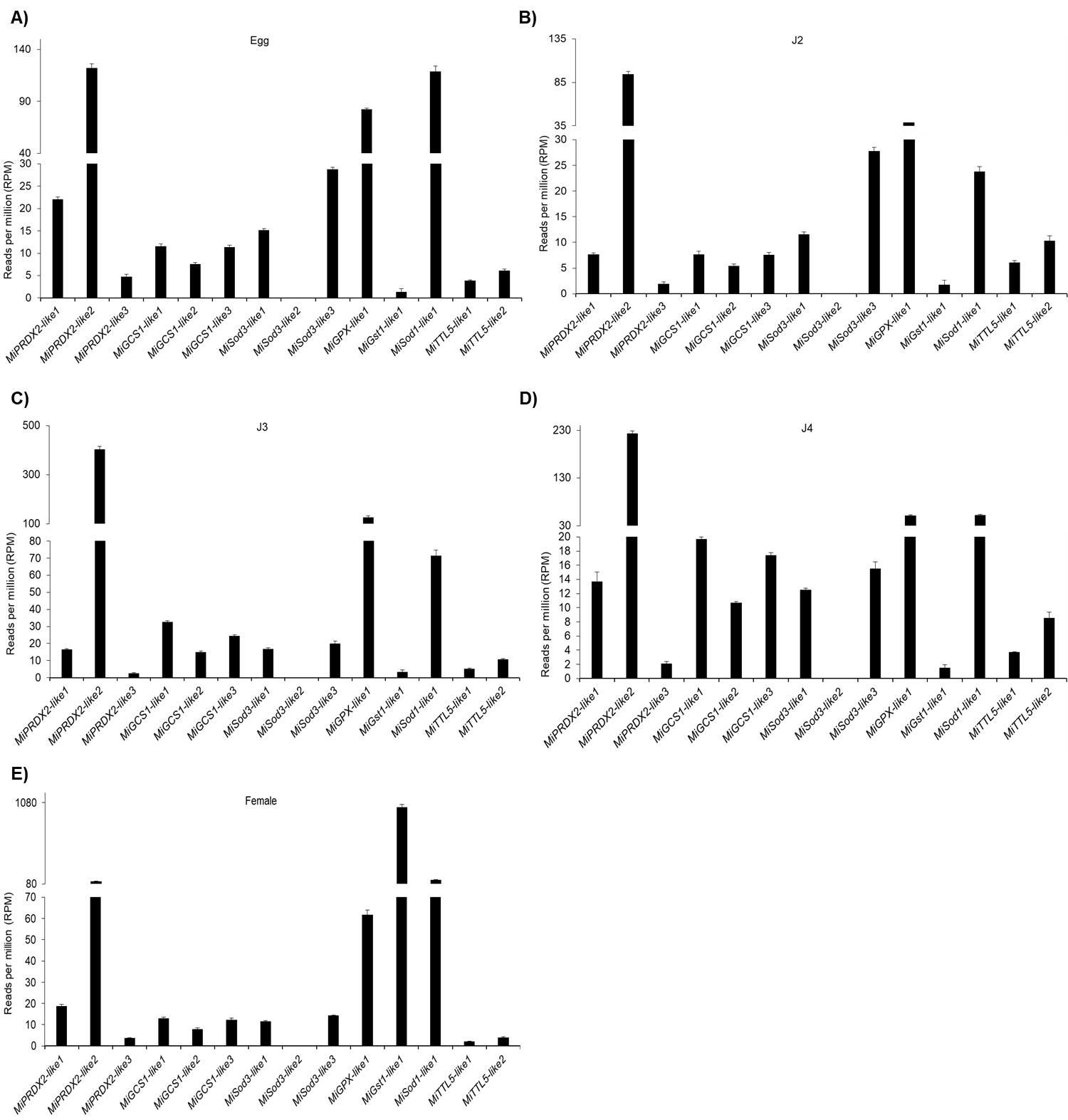


**Supplemental Figure 4.** Expression profiles of some defense genes from the DAF-16 and SKN-1 networks in **(A)** eggs, **(B)** J2, **(C)** J3, **(D)** J3, **(E)** J4, and **(F)** females of *Meloidogyne incognita* using transcriptome datasets (BioProject number: PRJNA390559; ^39^) retrieved from the BioSample database (NCBI). Error bars represent confidence intervals corresponding to three libraries per life stage of the nematode. The sequences of *MiPRDX2-like1* (Minc3s00535g13905), *MiPRDX2-like2* (Minc3s00365g11069), *MiPRDX2-like3* (Minc3s06441g39819), *MiGCS1-like1* (Minc3s00283g09347), *MiGCS1-like2* (Minc3s03748g34681), *MiGCS1-like3* (Minc3s00030g01824), *MiSod3-like1* (Minc3s03341g33565), *MiSod3-like2* (Minc3s03341g33566), *MiSod3-like3* (Minc3s01794g26348), *MiGPX-like1* (Minc3s00235g08236), *MiGst1-like1* (Minc3s00369g11129), *MiSod1-like1* (Minc3s08143g41941), *MiTTL5-like1* (Minc3s01149g21190), and *MiTTL5-like2* (Minc3s03007g32403) genes were retrieved from BioProject ID PRJEB8714 (sample: ERS1696677) ^38^ from the WormBase database version WBPS13 ^67^.

**
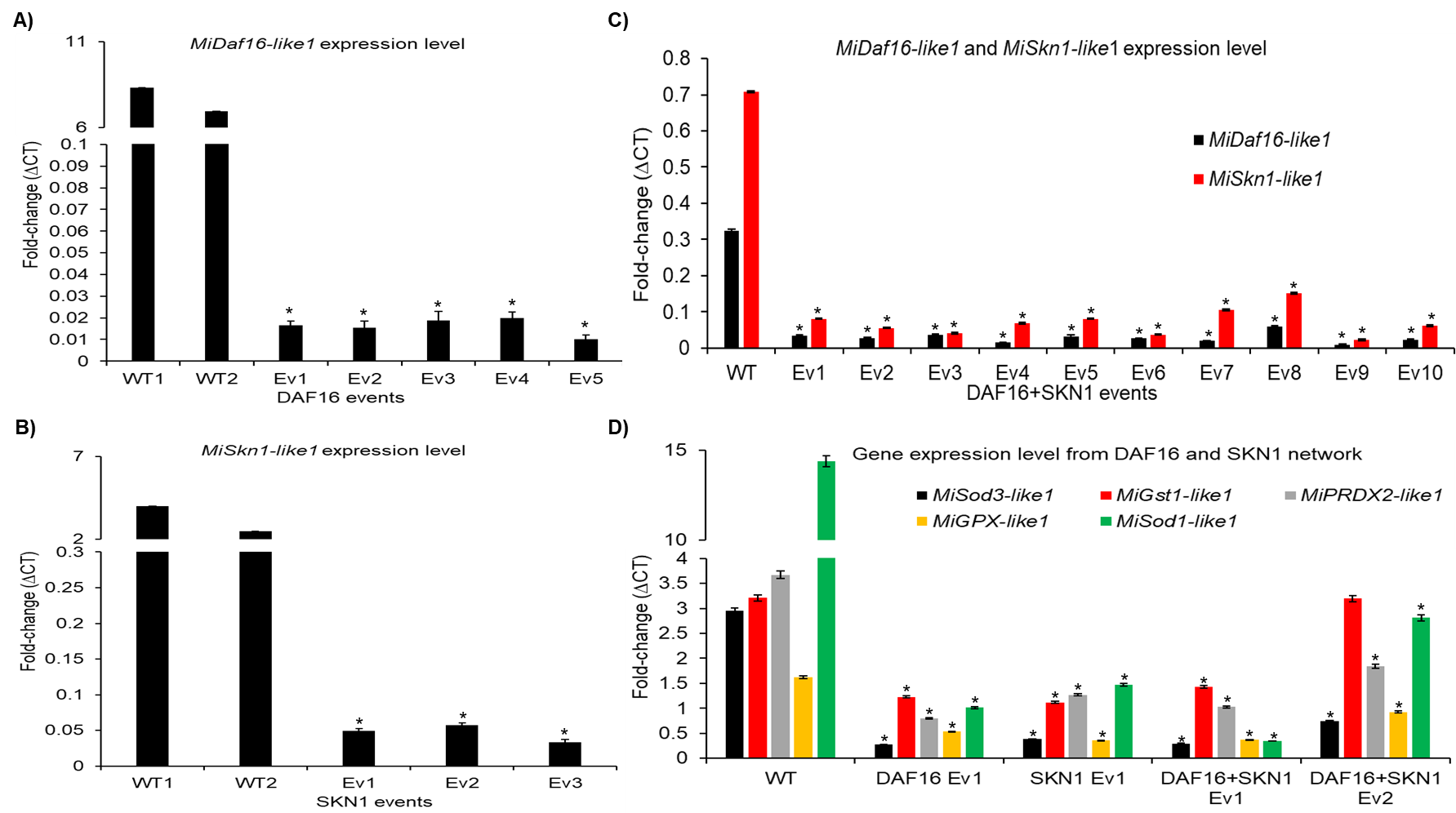
**

**Supplemental Figure 5.** Efficient downregulation of the *MiDaf16-like1* and *MiSkn1-like1* genes from *Meloidogyne incognita* race 3 during nematode infection in *Arabidopsis thaliana* transgenic plants. Expression profile of **(A)** *MiDaf16-like1* and **(B)** *MiSkn1-like1* genes in *M. incognita* during nematode infection in single-gene silencing plants. **(C)** Expression profile of *MiDaf16-like1* and *MiSkn1-like1* genes in *M. incognita* during nematode infection in double-gene silencing plants. **(D)** Expression profile of defense genes from the DAF16 and SKN-1 network in *M. incognita* during nematode infection in single- and double-gene silencing plants. Galls from five plants of each line were harvested at 60 days post-inoculation and processed in pool form. Galls harvested in *A. thaliana* wild-type (WT) were used as a positive control of the expression of the gene of interest. The fold change was calculated with the ∆CT formula using the *glyceraldehyde 3-phosphate dehydrogenase* (*MiGAPDH*) gene as the endogenous reference gene (Supplemental Table 3). Error bars represent confidence intervals corresponding to three technical replicates. Asterisks indicate significant differences based on Tukey’s test at 5%.

**REFERENCES**

23. Lin, B., et al., A novel nematode effector suppresses plant immunity by activating host reactive oxygen species-scavenging system. The New phytologist, 2016. 209(3): p. 1159-1173.

38. Blanc-Mathieu, R., et al. Hybridization and polyploidy enable genomic plasticity without sex in the most devastating plant-parasitic nematodes. PLoS genetics, 2017. 13, e1006777 DOI: 10.1371/journal.pgen.1006777.

39. Choi, I., et al., RNA-Seq of Plant-Parasitic Nematode *Meloidogyne incognita* at Various Stages of Its Development. Frontiers in Genetics, 2017. 8: p. 190-190.

67. Lee, R.Y N. et al. WormBase 2017: molting into a new stage. Nucleic Acids Research 46, D869-D874 (2017).

81. Marchler-Bauer, A. et al. CDD: NCBI's conserved domain database. Nucleic Acids Research 43, D222-226 (2015).

82. El-Gebali, S. et al. The Pfam protein families database in 2019. Nucleic Acids Research 47, D427-D432 (2018).

83. la Cour, T. et al. Analysis and prediction of leucine-rich nuclear export signals. Protein Engineering, Design and Selection 17, 527-536 (2004).

84. Nguyen Ba, A.N., Pogoutse, A., Provart, N. & Moses, A.M. NLStradamus: a simple Hidden Markov Model for nuclear localization signal prediction. BMC Bioinformatics 10, 202 (2009).
